# Quantum Dynamics Simulations of Exciton Polariton Transport

**DOI:** 10.1021/acs.nanolett.4c05674

**Published:** 2025-01-21

**Authors:** Benjamin X. K. Chng, M. Elious Mondal, Wenxiang Ying, Pengfei Huo

**Affiliations:** † Department of Physics, University of Rochester, Rochester, New York 14627, United States; ‡ Department of Chemistry, University of Rochester, Rochester, New York 14627, United States; § Institute of Optics, Hajim School of Engineering and Applied Sciences, University of Rochester, Rochester, New York 14627, United States; ∥ Center for Coherence and Quantum Optics, University of Rochester, Rochester, New York 14627, United States

**Keywords:** Polariton Transport, Ballistic Motion, Exciton Polariton, Light-Matter Interactions, Quantum Electrodynamics Simulations, Group Velocity Renormalization

## Abstract

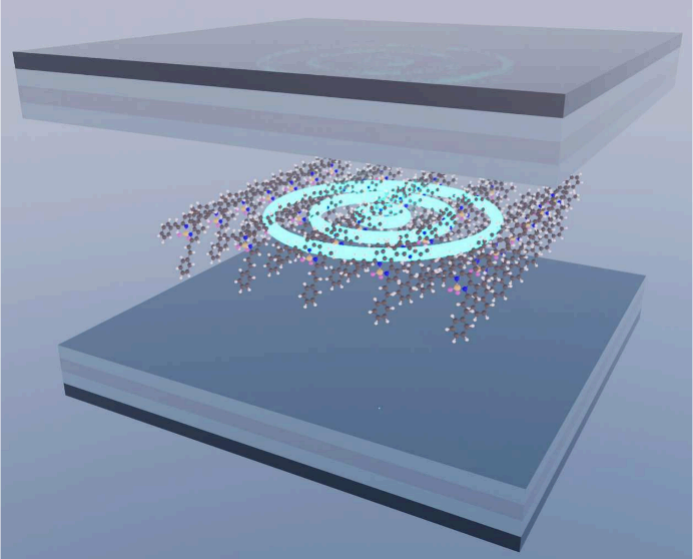

Recent experiments have shown that exciton transport can be significantly enhanced through hybridization with confined photonic modes in a cavity. The light-matter hybridization generates exciton-polariton (EP) bands, whose group velocity is significantly larger than the excitons. Dissipative mechanisms that affect the constituent states of EPs, such as exciton–phonon coupling and cavity loss, have been observed to reduce the group velocities in experiments. To elucidate the impacts of these dissipative mechanisms on polariton transport, we developed an efficient quantum dynamics approach that allows us to directly simulate polariton transport under the collective coupling regime and beyond long-wavelength approximation. Our numerical results suggest a renormalization of the group velocities with stronger exciton–phonon coupling strengths and a smaller Q-factor. We observe the transition from ballistic to diffusive propagation as well as the quality-factor-dependent behavior of the transient mean square displacement, agreeing well with the recent experimental measurements.

Enabling efficient excitation energy transport is essential for basic energy science and device applications. However, the inherent disorders and exciton–phonon interactions within these materials restrict transport, resulting in slow, diffusive motion of excitons that constrains the device’s performance. Recent experiments have demonstrated that exciton transport is significantly enhanced when they are strongly coupled to cavity modes.^1−5^ This leads to large group velocities up to 40 μm ps^–1^ for halide perovskites in a Fabry-Pérot (FP) cavity^3^ and up to 180 μm ps^–1^ for an organic semiconductor in a photonic crystal cavity^1^ with micrometer range of transport. In ref (1) the polariton transport achieves 100 μm in 1 ps, enabling long-range energy transport.

Experiments reveal that the measured group velocities of molecular polaritons are lower than those predicted by their dispersion relations,^1−3,6^ which is referred to as the renormalization of group velocity. Ultrafast measurements reported in refs (1) and (3) indicate more deviation from the predicted velocities with an increase in the wavepacket’s excitonic character. These findings underscore the importance of phonon-mediated scattering in influencing polariton transport via its excitonic component. Additionally, ref (2) reports that the polariton wavepacket’s velocities are strongly dependent on the cavity quality factor , although  is not related to the polariton’s dispersion. The -factor, which affects the polariton’s lifetime, also correlates with the polariton’s coherence lifetimes^7,8^ and hence, its transport properties, including both transient mean-square displacement (MSD) and group velocities.

We examine the influence of various dissipative mechanisms on the transport properties of molecular polaritons in an FP cavity using the mean-field Ehrenfest (MFE) quantum dynamics method^9,10^ to simulate the dynamics of the hybrid light-matter system. Notably, polariton transport occurs in a collective regime in which a substantial number of excitons (thousands or more) are resonantly coupled to cavity modes, resulting in upper polariton (UP) and lower polariton (LP) bands as well as dark states that do not contain any significant photonic character. To faithfully model the transport process, one needs to consider at least *N* = 10^4^ – 10^6^ molecules and  cavity modes (that satisfy the dispersion relation), presenting a significant computational challenge. Existing theoretical work either does not consider cavity loss^3^ or is limited to the size of the system^7^ in the transport simulations (such as only using *N* = 512 molecules and  modes in ref (7)).

We follow the previous work^3^ and consider the generalized Holstein-Tavis-Cummings (GHTC) Hamiltonian.^3,11−13^
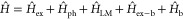
1The fundamental assumption used in a GHTC Hamiltonian (compared to the rigorous quantum electrodynamics Hamiltonian) can be found in ref (11) (see Sec. 2.6.1) as well as in refs (14−16). The excitonic Hamiltonian is *Ĥ*_ex_ = ∑_*n* = 0_^*N*–1^ (ℏω_ex_ + λ)σ̂_*n*_^†^σ̂_*n*_, where σ̂_*n*_^†^ = |*e*_*n*_⟩⟨*g*_*n*_| and σ̂_*n*_ = |*g*_*n*_⟩⟨*e*_*n*_| are the raising and lowering operators for the exciton on the *n*_th_ molecule, respectively. Further, |*g*_*n*_⟩ and |*e*_*n*_⟩ are the ground state and excited state of the *n*_th_ molecule, respectively, ℏω_ex_ = *E*_*e*_ – *E*_*g*_ is the excitation energy between the ground and excited states, and λ is the reorganization energy (that give rise to Stokes shift in linear spectra) due to exciton–phonon coupling. The *Ĥ*_ex-b_ + *Ĥ*_b_ terms further describe interactions between exciton and phonon bath, with details provided in the Supporting Information.

We model the FP cavity with an open direction *x* characterized by an in-plane wavevector *k*_∥_ and one confined direction *z* where *k*_⊥_ is the wavevector of the fundamental mode confined between two cavity mirrors, perpendicular to the mirror surface. The frequencies of the cavity mode are given by
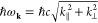
2where *c* is the speed of the light. When *k*_∥_ = 0, ℏω_**k**_(0) = ℏ*ck*_⊥_ ≡ ℏω_c_ is the cavity frequency at normal incidence. The photonic Hamiltonian *Ĥ*_ph_ is expressed as , and *â*_**k**_^†^ and *â*_**k**_ are the photonic raising and lowering operators for mode **k**, respectively. We consider *k*_∥_ with discrete (but still quasi-continuous) values , where the mode indexes , and  is the total number of cavity modes needed to capture the relevant energies for the hybrid system.

The light-matter interaction *Ĥ*_LM_ term is
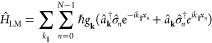
3where *x*_*n*_ is the location of the *n*_th_ molecule.^11^ Further, the *k*_∥_-dependent light-matter coupling strength is , where *g*_c_ is the single-molecule light-matter coupling strength at *k*_∥_ = 0 and is chosen as a parameter. A schematic of the model system is provided in Figure S1 of the Supporting Information. The transport dynamics occur in the single excitation subspace
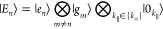
4a
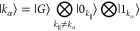
4bwhere |*E*_*n*_⟩ is the singly excited state for the *n*_th_ molecule located at *x*_*n*_, |*k*_α_⟩ is the 1-photon-dressed ground state with wave-vector *k*_∥_ = *k*_α_, and |*G*⟩ = ⊗_*n*_|*g*_*n*_⟩⊗_α_|0_*k*_α__⟩ represents the ground state. We assume identical loss rates Γ_c_ for all cavity modes *k*_α_, which is consistent with angle-resolved reflectance measurements of a typical FP cavity^17^ and previous theoretical work,^7^ and define the cavity quality factor at normal incidence (*k*_∥_ = 0) as .

We use an -MFE dynamics approach^9,10,18^ to simulate the polariton transport quantum dynamics in a lossy cavity. This approach describes the exciton-photonic degrees of freedom (DOF) quantum mechanically.
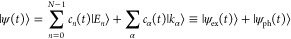
5The influence of phonons is computed using the Ehrenfest mixed quantum-classical dynamics, and cavity loss is computed through Lindblad dynamics using a stochastic approach,^9^ with details provided in the Supporting Information. The spatial distribution of the polariton is given by |ψ_±_(*x*_*n*_, *t*)|^2^ = |⟨±, *n*|ψ(*t*)⟩|^2^, with the real space polariton states expressed as
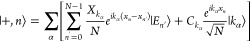
6a
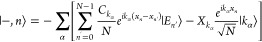
6bwhere *C*_*k*_α__ and *X*_*k*_α__ are the Hopfield coefficients^19^ at the in-plane momentum *k*_α_. Detailed derivations are provided in the Supporting Information.

To propagate quantum dynamics, we solve , where *Ĥ*_Q_ = *Ĥ* – *Ĥ*_b_ is the quantum part of the Hamiltonian (that include excitonic and photonic DOF). Solving it requires the operation of *Ĥ*_Q_ on |ψ(*t*)⟩, which is computationally expensive. We develop a novel computational algorithm by realizing that , where the ⊙ represents a simple Hadamard product between vectors, and  and  are Fast Fourier Transform (FFT) and inverse FFT, respectively. Further, |ϵ_ψ_⟩ represents a column matrix with the diagonal matrix element of *Ĥ*_Q_, with |ϵ_ψ_⟩ ≡ [{⟨*E*_*n*_|*Ĥ*_Q_|*E*_*n*_⟩}, {⟨*k*_α_|*Ĥ*_Q_|*k*_α_⟩}]^T^. This algorithm leads to a reduction in computational cost from  to  and a nearly 100 times speedup for *N* = 10^4^ molecule simulation, see details in the Supporting Information. All of the results presented in this work are performed under *T* = 300 K.

Figure 1 illustrates the impacts of reorganization energy λ (in panel b) and cavity loss rate Γ_c_ (in panel c) on polariton group velocities *v*_*g*_. Here, *v*_*g*_ is computed by following the wavefront of the polariton wavepacket, using the method outlined in ref (3), with details provided in the Supporting Information.

**Figure 1:**
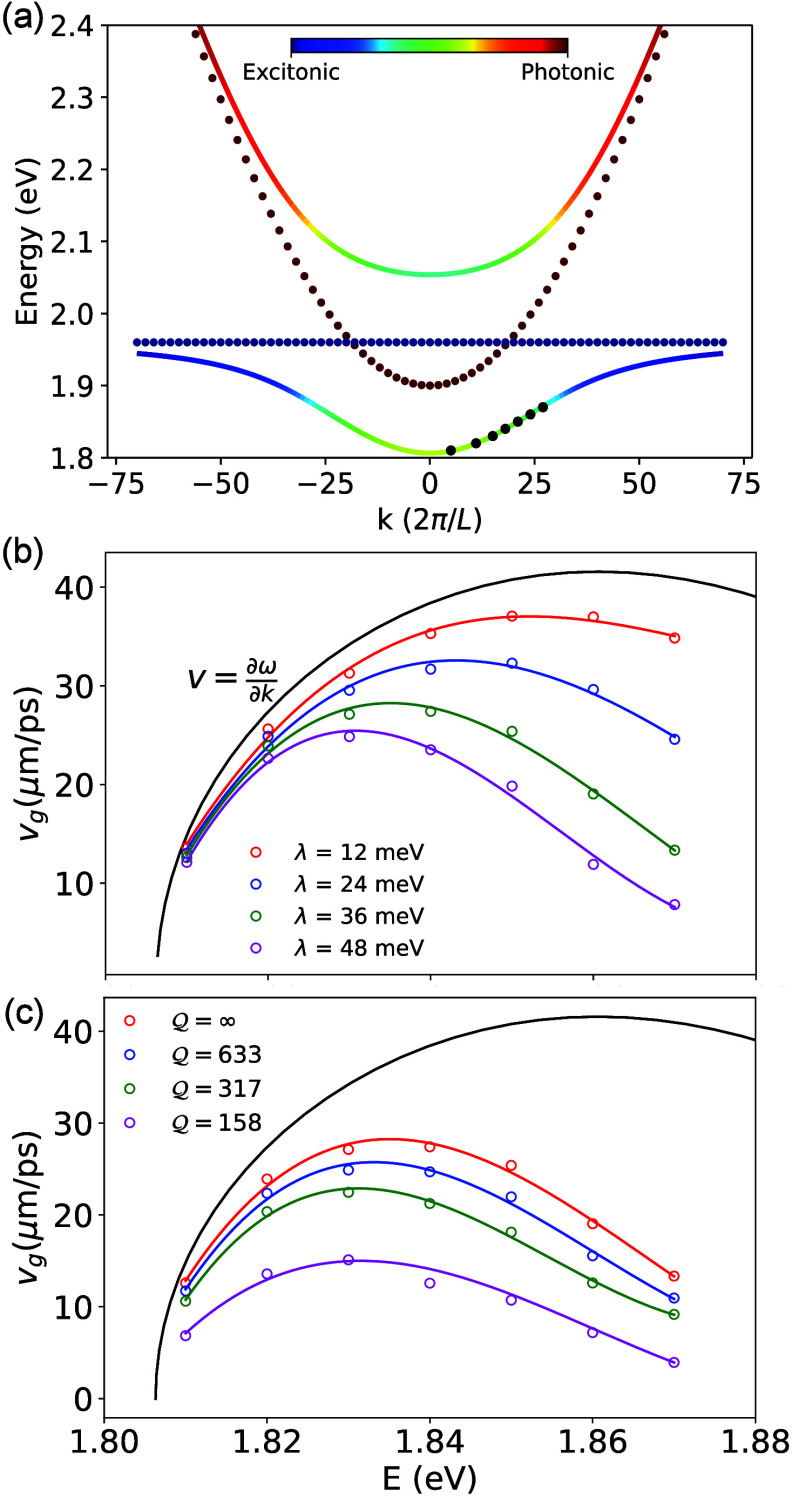
**Energy-resolved investigation of dissipative effects on polariton group velocity.** (**a**) Dispersion curve of the photon (red dots) and matter (blue dots). (**b**) Group velocities *v*_*g*_ of polaritons for various reorganization energy λ in a lossless cavity. (**c**) Group velocities *v*_*g*_ of polaritons for different cavity quality factor  with phonon reorganization energy λ = 36 meV.

Figure 1a presents the energy diagrams for the UP and LP bands formed by hybridizing the photonic band and the excitonic band. The LP and UP bands are color-coded based on their photonic character. The collective light-matter coupling strength is √*Ng*_c_ = 120 meV. These polariton states are analytically expressed in eq S13 in the Supporting Information. The initial excitation conditions are indicated in Figure 1 using black dots on the LP branch, corresponding to a pulse with a narrow energy bandwidth (to model the experimental condition in ref (3)).

Figure 1b shows *v*_*g*_ with different initial energies (corresponding to different *k*_∥_ values in Figure 1a). Here, the cavity is lossless with Γ_c_ = 0. The solid black line indicates the group velocity obtained as *v*_*g*_ = ∂ω_–_/∂*k*_∥_. The open circles with different colors are *v*_*g*_ for the excitonic system with different reorganization energy λ. As λ increases, *v*_*g*_ decreases, indicating that the polaritons propagate at a reduced *v*_*g*_ due to increased exciton–phonon coupling, which was referred to as the group velocity renormalization.^3^ Increasing λ or increasing the excitonic character (increasing *E*) causes more renormalization of *v*_*g*_, in agreement with the results in ref (3). Figure 1c illustrates the effects of cavity loss on *v*_*g*_ by changing the  factor (loss rate Γ_c_), with fixed λ = 36 meV. With a decreasing  (increasing Γ_c_), *v*_*g*_ further decreases due to rapid attenuation of the photonic contribution to the polariton wavepacket.

Figure 2a presents the impact of cavity quality factor  on *v*_*g*_ with a broadband excitation on the UP band (indicated with the gray Gaussian wavepacket in the inset of Figure 2a), to model similar experimental conditions in ref (2). Here, we use λ = 36 meV and vary the  factor. As shown in Figure 2, *v*_*g*_ increases with increasing . Our results demonstrate a trend consistent with experimental measurements in ref (2) (see Figure 2e of that work).

**Figure 2:**
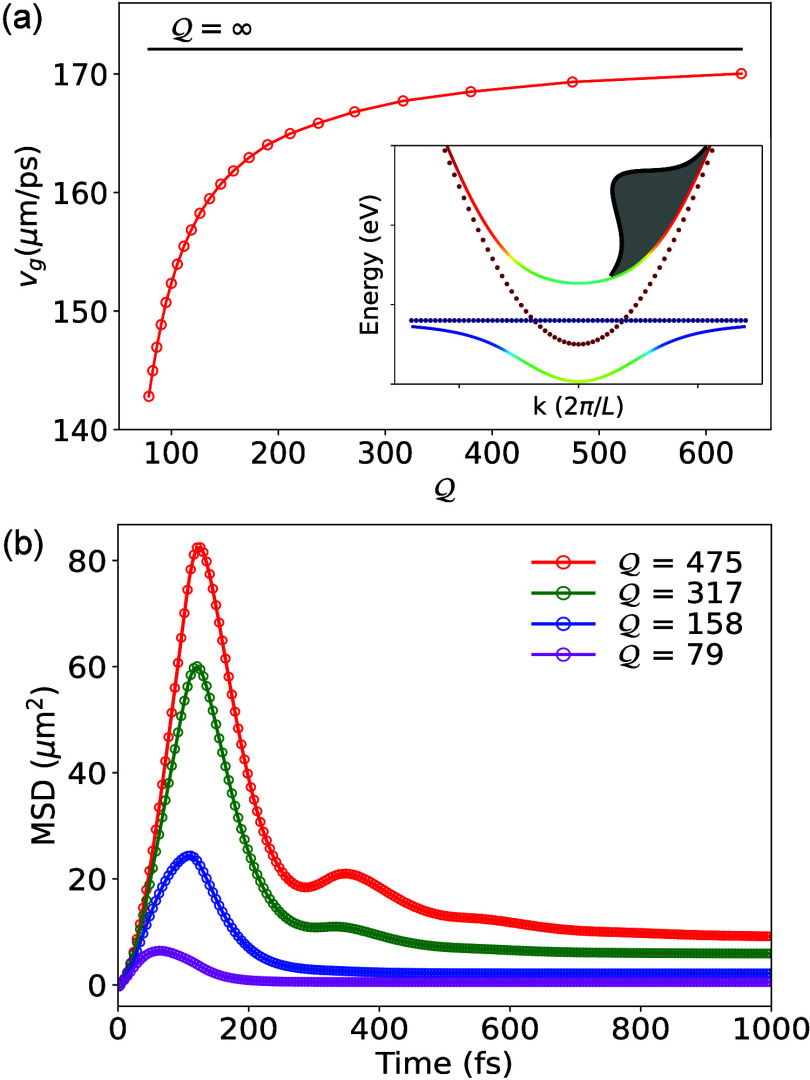
**Group velocity dependence on quality factor for UP broadband excitation.** (**a**) Polariton group velocity *v*_*g*_ vs cavity quality factor . The inset shows the energy bandwidth used for the initial excitation in the UP branch, optimizing the localization of the polariton wavepacket. Results are converged with 250 trajectories, validated against a set of runs with 1000 trajectories. (**b**) Time-dependent transient MSD with UP initial excitation for various .

Figure 2b presents the transient MSD σ^2^(*t*) of a polariton wavepacket under various -factors, which is computed as^7,20^
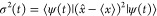
7where ⟨*x*⟩ is the centroid of the initial polariton wavepacket (at *t* = 0) in position space. In a very lossy cavity  = 79 (magenta curve), the wavepacket shows minimal spread within a brief duration of time for *t* ∼ 50 fs, and its MSD returns to its initial value after a long time (∼1 ps). With an increase in , both the wavepacket’s maximum MSD and the corresponding rise time increase. The initial rise of MSD is again attributed to the polariton’s photonic character, which is responsible for ballistic transport, as pointed out in recent theoretical work.^7^ The dip in MSD right after the initial rise is attributed to both the decay of the UP population to the dark states and to cavity loss, which are competing at a similar time scale, with details provided in Figure 3. We note that the steady-state MSD in lossy cavities with  surpasses the MSD of the original polariton wavepacket at *t* = 0, suggesting that strong light-matter coupling (here, √*Ng*_c_ = 120 meV) facilitates the expansion of the underlying excitons over a larger volume even in the presence of cavity loss. The observed trends in our numerical simulations are consistent with transient absorption spectral measurements (see Figure 2c in ref (2)). Similar studies in which the LP branch is excited are reported in the Supporting Information, and we observe a similar trend for the transient MSD but no corresponding rise and dip.

**Figure 3:**
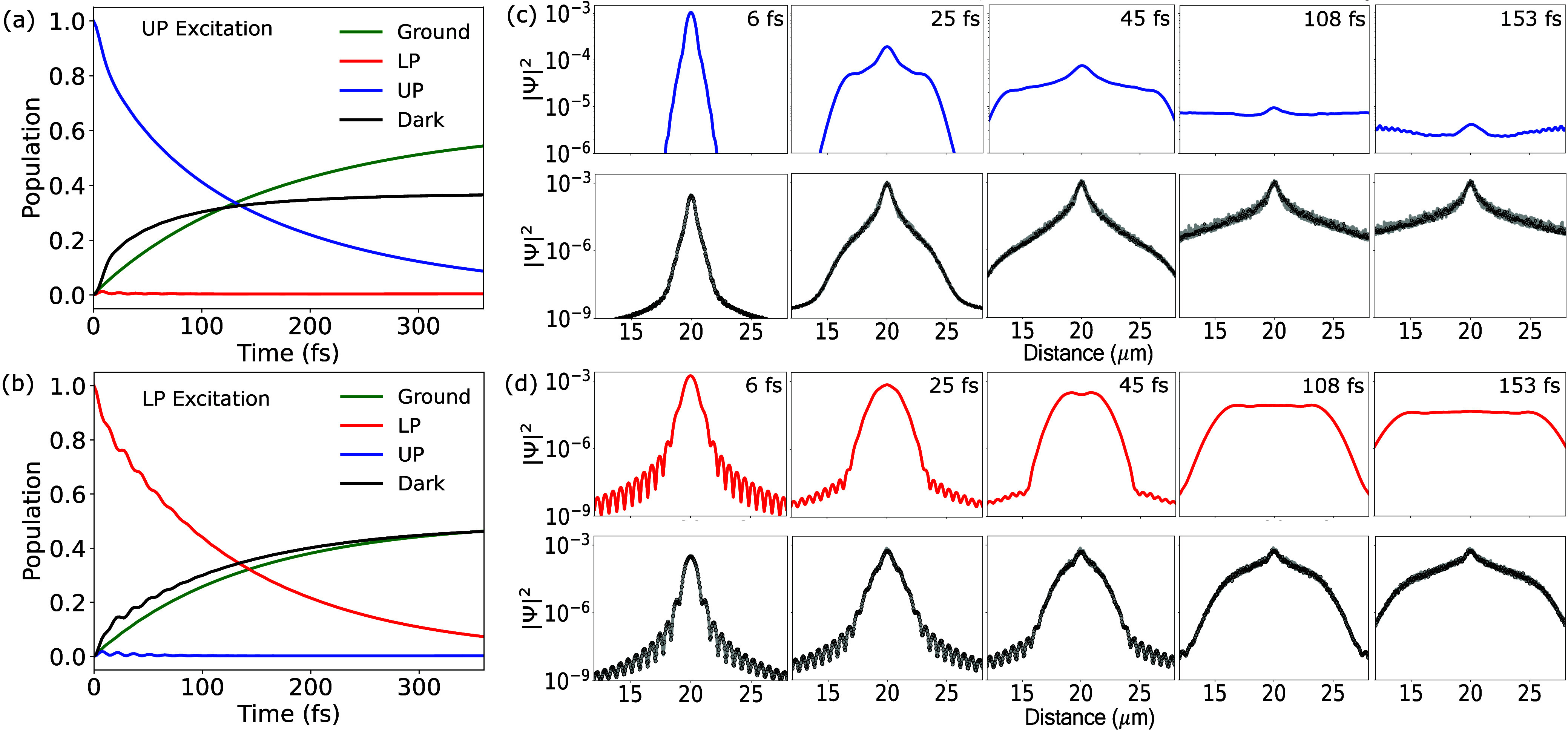
**Polariton population dynamics and wavepacket.** The populations of UP (blue), LP (red), dark (black), and ground states (green) in a cavity with  are presented, with (**a**) broadband UP excitation and (**b**) broadband LP excitation. The wavepackets in position space, decomposed into polariton and dark-state components, are illustrated for (**c**) broadband UP excitation and for (**d**) broadband LP excitation.

Figure 3a and Figure 3b present the population dynamics of the UP (blue), LP (red), and dark states (black) under broadband UP excitation and broadband LP excitations, respectively, with cavity quality factors of . The ground-state population (green) is also depicted. For *N* molecules and  cavity modes, there is a total of  different UP states (with different *k*_α_),  different LP states, and  dark exciton states. The definitions of UP, LP, and Dark states are provided in eqs S12–S13 of the Supporting Information. Here, we group the same types of states together to visualize the transitions among these manifolds of states.

In Figure 3a, an initial substantial decrease in the UP population is observed, accompanied by a sharp increase in the dark-state population and a slower rate of increase in the ground-state population. The population transfer from the UP to the dark state is mediated by exciton–phonon coupling,^21,22^ and the population transfer to the ground state is attributed to cavity loss. Here, with a high , it is evident that exciton–phonon coupling has more influence on the decay of the initial UP population than cavity loss. For the broadband LP excitation shown in Figure 3b, an initial substantial decrease in the LP population is observed, and the rate of population transfer from LP to the ground state is similar to the rate of population transfer from LP to the dark states.

Figure 3c,d presents the polariton wavepacket and the dark exciton density in the position space for broadband excitations. Over time, the UP (blue) and LP (red) wavepackets propagate outward from the center, primarily due to their *photonic character*, which exhibits ballistic transport (with *v*_*g*_ largely adopted from the derivative of the band). Due to the exciton–phonon coupling, the UP and LP wavepackets transfer population to the dark state, resulting in an increase in the dark state (black) probability densities. The resultant dark-state wavepacket is largely immobile, as it consists of excitons that move diffusively in most transport experiments. In the current theoretical studies, these dark states are completely immobile because of their dispersionless band assumed in the model (Figure 1a). However, we note that in some materials, even excitons exhibit wavelike transport at room temperature,^23^ and the role of the dark states need to be carefully examined for those materials when coupling them inside the cavity.

Due to the larger *v*_*g*_ of the UP wavepacket, the polariton wavepacket expands rapidly in early time, contributing to a sharp rise in the total transient MSD (Figure 2b). As the UP expands, it also transfers populations to the dark exciton states (at the location of *x*_*n*_ which UP is visiting) but with a slower transfer speed compared to *v*_*g*_. Consequently, the UP wavepacket (blue curve in Figure 3c) propagates more rapidly than the dark state wavepacket (black curve in Figure 3c). Note that the dark exciton in our model has *v*_*g*_ = 0 from the derivative of the dispersion curve. The effective propagation of the dark exciton wavepacket is only due to the transfer of population from UP that propagates ballistically in space at an early time. After *t* = 108 fs, the UP wavepacket is further spread out and its amplitude also starts to decrease due to cavity loss, resulting in a decrease in its magnitude, as well as its contribution to the transient MSD. In addition, the interference term for UP excitation is constructive, further adding to the contribution of transient MSD at an early time. This constructive interference is fragile to decoherence and eventually disappears after a long time. Thus, a faster *v*_*g*_ in the UP band, a relatively slow UP to dark-state transition rate, and constructive interference between exciton and photon wavepacket, together explain the MSD behavior as shown in Figure 2b, where the MSD initially increases to a peak value before declining.

In contrast, for the LP wavepacket, Figure 3d shows a more gradual expansion of the polariton wavepacket. For instance, at *t* = 45 fs, the LP wavepacket (red curves) spans a width ranging from about 18 to 22 μm only, which is similar to the width of the corresponding dark-state wavepacket at *t* = 45 fs. This is because the LP wavepacket advances at a rate comparable to the rate of LP to dark-state transition, resulting in a synchronized expansion of both LP and dark exciton wavepacket. In addition, the interference contribution for MSD is destructive, which further reduces the transient MSD (see Figure S3 in Supporting Information).

We investigate the behavior of polariton transport due to the influence of the photonic character of the initial wave packet at *t* = 0. We consider λ = 36 meV,  (which is in line with the cavity used in ref (1)), and a narrow band of initial excitation conditions with a narrow range of *k*_∥_ on the LP state (see Figure 1a), to model the *k*-selective probing conditions in ref (3) and ref (1). Here, we consider a cavity with a lower frequency for this study (ℏω_*c*_ = 1.77 eV at *k*_∥_ = 0), which is more red-detuned compared to the curve presented in Figure 1a. For each initial excitation condition with a given *k*_∥_, we report the corresponding photonic characters |χ_ph_|^2^ = ∑_α_|*c*_α_(*t* = 0)|^2^ of the wavepacket, defined as the sum of the photonic components of the polariton at *t* = 0. To determine the transport characteristics, we perform a least-squares fitting of the transient MSD with the equation
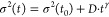
8which corresponds to a generalized diffusion equation.^1,24^ The constant *D* represents the generalized diffusion coefficient, while the exponent γ characterizes the transport properties. For γ = 1, the transport is diffusive, for γ = 2, the transport is ballistic,^1,3^ and for γ < 1, the transport is subdiffusive.^25^

Figure 4a presents the time-dependent MSD (eq 7). We find that there are two separate transport stages, one at early times with γ ≈ 2 (with black solid lines as the fitting lines) and one at later times, with γ ≈ 1 (red solid lines as fitting lines). Figure 4b provides the value of γ as a function of |χ_ph_|^2^, obtained from the fitting in panel (a). The duration of the ballistic stage depends on the photonic character of the wavepacket at *t* = 0. For small photonic characters (|χ_ph_|^2^ = 0.45), the ballistic stage lasts for up to 50 fs, while for large photonic characters (|χ_ph_|^2^ = 0.75), the ballistic stage lasts for a longer duration of up to 100 fs. Further, the wavepacket transitions from purely ballistic (|χ_ph_|^2^ = 0.75) to purely diffusive (|χ_ph_|^2^ = 0.35) transport as |χ_ph_|^2^ decreases. This suggests that for the initial polariton wavepacket, the transport is ballistic for a duration (with *v*_*g*_ being renormalized by exciton–phonon coupling and cavity loss) before gradually becoming diffusive. This observation is in close agreement with recent experiments (e.g., Figure 2c in ref (3) and Figure 4 in ref (1)).

**Figure 4:**
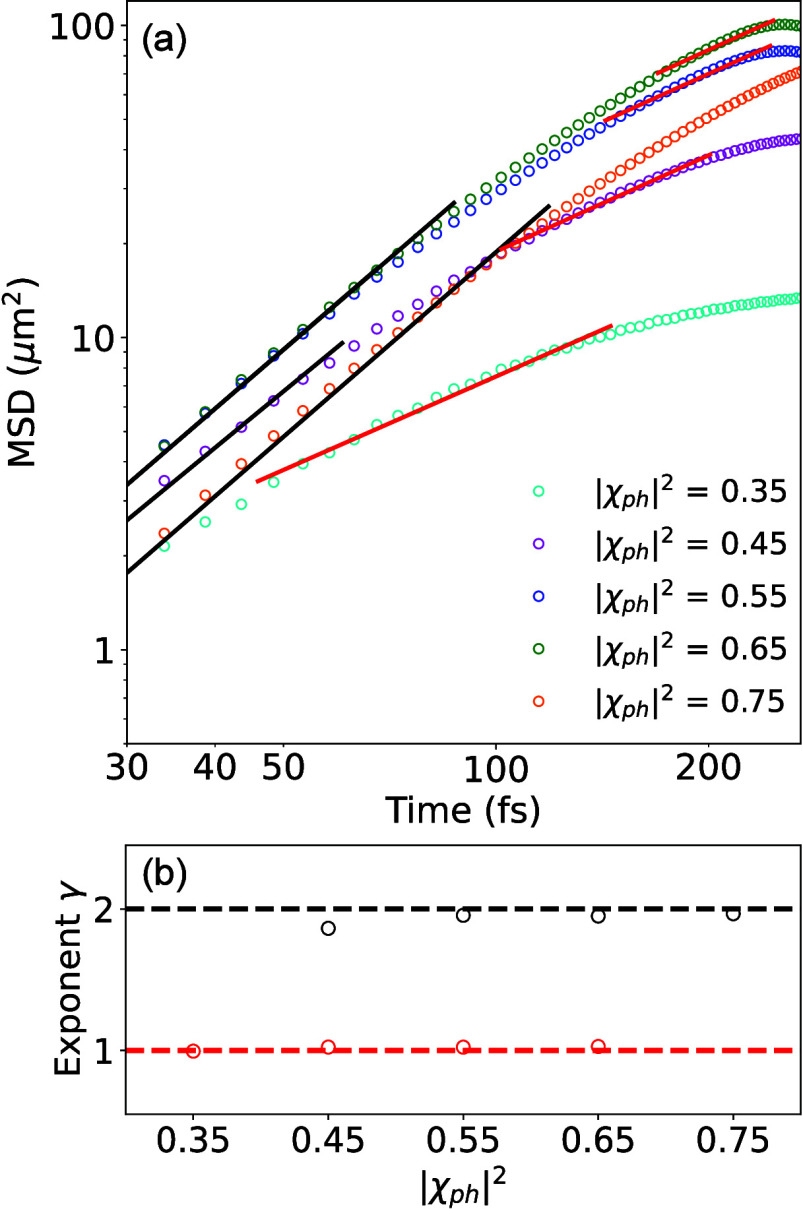
**A transition from ballistic to the diffusive transport.** (**a**) MSD for the polariton as a function of time, with various photonic character |χ_ph_|^2^. (**b**) The exponents extracted from fitting the MSDs with various photonic character |χ_ph_|^2^ with eq 8.

Note that even under the diffusive transport stage (γ ≈ 1) the group velocity is still much larger than the expected gradient of matter dispersion (which is 0 in the current model). Also, the absolute value of MSD depends on the group velocity, and with a large photonic contribution |χ_ph_|^2^ = 0.75, the gradient of the LP dispersion is relatively small (close to a small *k*_∥_ value in the FP cavity as indicated in Figure 1a). As such, for |χ_ph_|^2^ = 0.75, even though the transport is ballistic for the longest time (for *t* < 100 fs) compared to the other cases investigated here, the MSD is not necessarily the largest.

We developed an efficient approach for investigating polariton transport with quantum dynamics simulations. We achieved quasi-linear scaling for our quantum dynamical method, enabling simulations of *N* = 10^4^ molecules collectively coupled to  cavity modes in a GHTC Hamiltonian. The results from quantum dynamics simulations confirm the *v*_*g*_ renormalization effects^1,3^ and demonstrate the *v*_*g*_ reduction due to exciton–phonon coupling and cavity loss. Furthermore, the transient MSD of polariton wavepackets with broadband UP excitation demonstrates transient growth and then contraction, agreeing with the experimental observations in ref (2). This is due to the fast expansion of the UP polariton wavepacket in space and the relatively slower rate of transitions to the dark exciton wavepacket, as demonstrated by our quantum dynamics analysis (Figure 3). Finally, from the transient MSD, we were able to analyze the transport characteristics of the wavepacket that illustrates a ballistic-to-diffusive turnover, which has been experimentally observed in ref (3) and ref (1). Overall, the results from our quantum dynamics simulations successfully capture all the trends observed in recent polariton transport experiments.^1−3^ The current theory does not consider static disorders,^26,27^ intermolecular interactions, or Peierls phonon (that fluctuates the intersite couplings). Their influence will be explored in future work.

## Supporting Information Available

Details of Model Hamiltonian; Polariton Quantum Dynamics Propagation Method; Details of Quantum Dynamics Simulations; Details of Polariton Transport Properties Calculations; Additional Numerical Results (PDF)
