# Supplementary material for: Quantum Dynamics Simulations of Exciton Polariton Transport
Source: Nano Lett. 2025 Jan 21;25(4):1617–22. doi: 10.1021/acs.nanolett.4c05674 (PMC11783598; doi:10.1021/acs.nanolett.4c05674)
Supplement: Supplementary file 1 — nl4c05674_si_001.pdf [file nl4c05674_si_001.pdf]

Supporting Information:

Quantum Dynamics Simulations of Exciton Polariton Transport

Benjamin X. K. Chng,[†] M. Elious Mondal,[‡] Wenxiang Ying,[‡] and Pengfei Huo[*,‡,¶,§]

†*Department of Physics and Astronomy, University of Rochester, Rochester, NY 14627, U.S.A.*

‡*Department of Chemistry, University of Rochester, Rochester, NY 14627, U.S.A.*

¶*The Institute of Optics, Hajim School of Engineering, University of Rochester, Rochester, NY 14627, U.S.A.*

§*Center for Coherence and Quantum Optics, University of Rochester, Rochester, New York 14627, USA*

E-mail: pengfei.huo@rochester.edu

I. Details of the Model Hamiltonian

Generalized Holstein-Tavis-Cummings Hamiltonian.

We use the generalized Holstein-Tavis-Cummings (GHTC) Hamiltonian[1–3] to describe N molecules collectively coupled to multiple cavity modes as follows

$$\hat{H} = \hat{H}_{\text{ex}} + \hat{H}_{\text{b}} + \hat{H}_{\text{ex-b}} + \hat{H}_{\text{ph}} + \hat{H}_{\text{LM}}, \tag{S1}$$

where $\hat{H}_{\text{ex}}$ is the excitonic Hamiltonian describing N non-interacting molecules, $\hat{H}_{\text{b}}$ is the molecular bath Hamiltonian describing the phonon modes associated with each molecule, $\hat{H}_{\text{ex-b}}$ is the exciton-phonon interaction, $\hat{H}_{\text{ph}}$ is the photonic Hamiltonian describing the electromagnetic modes in a cavity, and $\hat{H}_{\text{LM}}$ is the light-matter interaction term.

The photonic Hamiltonian $\hat{H}_{\text{ph}}$ is expressed as[2–4]

$$\hat{H}_{\text{ph}} = \sum_{\text{k}_{\parallel}} \hbar\omega_{\mathbf{k}}(\hat{a}^{\dagger}_{\mathbf{k}}\hat{a}_{\mathbf{k}} + \frac{1}{2}), \tag{S2}$$

where $\mathbf{k}$ is the wave vector corresponding to a given cavity mode. Here, we analyze a one-dimensional cavity, with a quasi-continuous open direction x characterized by an in-plane wavevector $k_{\parallel}$, and a confined direction z where $k_{\perp}$ is the wavevector of the fundamental mode confined between two cavity mirrors, perpendicular to the mirror surface. Consequently, the frequencies of the cavity mode are given by

$$\hbar\omega_{\mathbf{k}} = \hbar c\sqrt{k_{\parallel}^2 + k_{\perp}^2}, \tag{S3}$$

where c is the speed of the light and we assumed the reflective index inside the cavity is $n_{\text{c}} = 1$. When $k_{\parallel} = 0$, $\hbar\omega_{\mathbf{k}}(0) = \hbar k_{\perp} = \hbar\omega_{\text{c}}$ which is the typical cavity frequency for a single-mode approximation.

Further, we assume there are N molecules equally spaced a distance L apart (lattice constant) along the $k_{\parallel}$ direction inside the cavity, and we assume periodic boundary conditions[2] along the $k_{\parallel}$ direction, such that

$$k_{\alpha} \equiv k_{\parallel}(\alpha) = \frac{2\pi}{NL}\alpha \tag{S4}$$

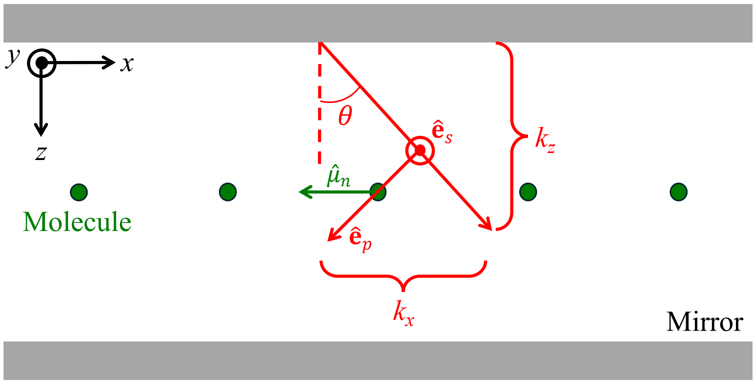

Figure S1: A Schematic illustration of many molecules coupled inside a Fabry-Perot (FP) cavity. Here $\hat{\mathbf{e}}_{\mathrm{s}}$ and $\hat{\mathbf{e}}_{\mathrm{p}}$ are the unit vectors for the s (transverse electronic, TE) and p polarizations (Transverse Magnetic, TM) respectively. We assumed the molecules all have the same dipole moments $\hat{\mu}_n$ oriented along the $-x$ direction. Here, $k_{\|} = k_x$, and $k_{\perp} = k_z$.

where the mode index is $\alpha \in [-\frac{\mathcal{M}}{2}, ...0, ...\frac{\mathcal{M}}{2}]$, and $\mathcal{M}$ is the total number of cavity modes needed to capture the relevant energies for the hybrid system.

The excitonic Hamiltonian $\hat{H}_{\mathrm{ex}}$ is given by the expression

$$\hat{H}_{\mathrm{ex}} = \sum_{n=0}^{N-1} (\hbar\omega_{\mathrm{ex}} + \lambda)\hat{\sigma}_n^{\dagger}\hat{\sigma}_n, \quad \text{(S5)}$$

where $\hbar\omega_{\mathrm{ex}} = E_e - E_g$ is the excitation energy between the ground and excited states, λ is the reorganization energy due to exciton-phonon coupling. Furthermore, $\hat{\sigma}_n^{\dagger}$ and $\hat{\sigma}_n$ correspond to the raising and lowering operators for the excitons (electronic degrees of freedom (DOF)). We consider a linear chain of molecules, each centered at x_n, with a uniformly spacing $L = x_n - x_{n-1}$, and satisfied the boundary condition $x_N = x_0$, forming a simulation box of size NL.

The light-matter interaction $\hat{H}_{\mathrm{LM}}$ term is expressed as[1–4]

$$\hat{H}_{\mathrm{LM}} = \sum_{k_{\|}} \sum_{n=0}^{N-1} g_{\mathbf{k}} \left(\hat{a}_{\mathbf{k}}^{\dagger}\hat{\sigma}_n e^{-ik_{\|}x_n} + \hat{a}_{\mathbf{k}}\hat{\sigma}_n^{\dagger} e^{ik_{\|}x_n} \right), \quad \text{(S6)}$$

where $x_n = n \cdot L$ is the location of the n_{th} molecule, and the $k_{\|}$-dependent light-matter coupling strength $g_{\mathbf{k}}$ in Eq. S6 is

$$g_{\mathbf{k}} = \hbar g_{\mathrm{c}} \sqrt{\frac{\omega_{\mathbf{k}}(k_{\|})}{\omega_{\mathbf{k}}(0)}} \cos\theta, \quad \text{(S7)}$$

where g_c is the single-molecule coupling strength, and $\tan\theta = k_{\|}/k_{\perp}$, and $\cos\theta = |\hat{\mu} \cdot \hat{\mathbf{e}}|$ account

for the relative dipole orientation with respect to the field polarization. Here, we consider the configuration of dipoles coupled to the TM polarization (p-polarization), as shown in Fig. S3 with the relative angle θ. One can instead, also consider the dipole orientation to be perpendicular to the paper and perfectly align with the TE polarization (s-polarization), such that all $\cos\theta = 1$ regardless of the value of $k_{\parallel}$. In a typical experiment[3] of polariton transport in the FP cavity, the matter will couple to both polarizations.

Further, each exciton is coupled to its own phonon bath modes, with the bath Hamiltonian described by

$$\hat{H}_{\mathrm{b}} = \sum_{n=0}^{N-1}\sum_{\nu}\left(\frac{1}{2}\hat{P}_{n,\nu}^{2} + \frac{1}{2}\omega_{\nu}^{2}\hat{R}_{n,\nu}^{2}\right), \tag{S8}$$

and the system-bath (exciton-phonon) coupling term described by

$$\hat{H}_{\mathrm{sb}} = \sum_{n=0}^{N-1}\hat{\sigma}_n^{\dagger}\hat{\sigma}_n \otimes \sum_{\nu} C_{n,\nu}\hat{R}_{n,\nu}. \tag{S9}$$

Here, ω_{ν} is the frequency for the ν_{th} phonon mode, $\hat{R}_{n,\nu}$ and $\hat{P}_{n,\nu}$ are the position and momentum operators for the ν_{th} vibrational mode in the n_{th} molecule. The bath coupling constants, $c_{n,\nu}$, are models as the Debye spectral density[5]

$$\mathcal{J}(\omega) = \frac{\pi}{2}\sum_{\nu}\frac{C_{n,\nu}^{2}}{\omega_{n,\nu}}\delta(\omega - \omega_{n,\nu}) = \frac{2\lambda\omega_{\mathrm{b}}\omega}{\omega_{\mathrm{b}}^{2} + \omega^{2}}, \tag{S10}$$

where λ is the reorganisation energy as presented in Eq. S5, and ω_{b} is the characteristic frequency of the phonon bath. Further, λ can be formally defined as

$$\lambda = \frac{2}{\pi}\int_{0}^{\infty} d\omega \frac{\mathcal{J}(\omega)}{\omega} = \sum_{\nu}\frac{C_{n,\nu}^{2}}{\omega_{n,\nu}^{2}}. \tag{S11}$$

For the $\mathcal{L}$-MFE simulations, we sample Eq. S10 by using a total of $N_{\nu} = 35$ bath modes (for each molecule) for various values of λ meV, and $\omega_b = 6.2$ meV. The bath parameters are sampled

using the procedure outlined in Ref. 6 as follows

$$C_{n,\nu} = 2\sqrt{\lambda\frac{\tan^{-1}(\omega_{\max}\tau_\nu)}{\pi N_\nu \omega_\nu}}, \tag{S12a}$$

$$\omega_\nu = \frac{1}{\tau_\nu}\tan\left(\frac{\nu}{N_\nu}\tan^{-1}(\omega_{\max}\tau_\nu)\right), \tag{S12b}$$

where $\tau_\nu = 1/\omega_\mathrm{b}$, $\omega_{\max} \gg \omega_\mathrm{b}$ is the maximum frequency when discretizing the bath frequencies. Here, we choose $\omega_{\max} = 20\omega_\mathrm{b}$.

Polaritonic States and Dark States.

The polaritonic states are defined from the GHTC Hamiltonian without the bath and exciton-phonon terms. Here, we consider the Hamiltonian $\hat{H}_\mathrm{pl}$ as follows

$$\hat{H}_\mathrm{pl} = \hat{H}_\mathrm{ex} + \hat{H}_\mathrm{ph} + \hat{H}_\mathrm{LM}, \tag{S13}$$

which excludes the exciton-phonon coupling $\hat{H}_\mathrm{ex-b}$ in the polariton Hamiltonian, as opposed to the previous work that includes them in the definition of the polariton Hamiltonian.[3,7]

We define the bright states $|\mathrm{B}_\alpha\rangle$ and dark states $|\mathrm{D}_\kappa\rangle$ of the collective matter exciton as

$$|\mathrm{B}_\alpha\rangle = \frac{1}{\sqrt{N}}\sum_{n=0}^{N-1}\exp\Big(-2\pi i\frac{n\alpha}{N}\Big)|E_n\rangle = \frac{1}{\sqrt{N}}\sum_{n=0}^{N-1}\exp(-ik_\alpha x_n)|E_n\rangle, \quad \alpha \in \{-\frac{\mathcal{M}}{2}, \cdots, \frac{\mathcal{M}}{2}\}, \tag{S14a}$$

$$|\mathrm{D}_\kappa\rangle = \frac{1}{\sqrt{N}}\sum_{n=0}^{N-1}\exp\Big(-2\pi i\frac{n\kappa}{N}\Big)|E_n\rangle = \frac{1}{\sqrt{N}}\sum_{n=0}^{N-1}\exp(-ik_\kappa x_n)|E_n\rangle, \tag{S14b}$$

where the κ index for the dark states is

$$\kappa \in \left\{-\frac{(N+\mathcal{M})}{2}+1, \cdots, -\left(\frac{\mathcal{M}}{2}+1\right)\right\}, \quad \text{and } \kappa \in \left\{\frac{\mathcal{M}}{2}+1\cdots, \frac{(N+\mathcal{M})}{2}-1\right\},$$

and there are a total of $N-\mathcal{M}$ of dark states. Further, $k_\alpha = 2\pi\alpha/(NL)$, $k_\kappa = 2\pi\kappa/(NL)$, and $x_n = nL$. The value κ does not have a matching α index, and thus, these dark states will not mix with the photonic DOF with k_α. Further, we set the indices in Eq. S14b such that the α_th bright state couples to a photon mode with wave-vector k_α. Additionally, we define dark states $|\mathrm{D}_\kappa\rangle$ with indices κ that are greater than $\pm\mathcal{M}/2$ (c.f. Eq. S14b).

Due to the light-matter coupling, the bright states $|\mathrm{B}_\alpha\rangle$ hybridize with the photonic states. In the current model, there are a total of $\mathcal{M}$ photonic states $\{|k_\alpha\rangle\}$ and a total of $\mathcal{M}$ bright exciton states (Eq. S14b). The hybridization of bright exciton states and photonic states generates a total of $2\mathcal{M}$ upper and lower polariton states, expressed as

$$|+,k_\alpha\rangle = \cos\Theta_N|\mathrm{B}_\alpha\rangle + \sin\Theta_N|k_\alpha\rangle, \tag{S15a}$$

$$|-,k_\alpha\rangle = -\sin\Theta_N|\mathrm{B}_\alpha\rangle + \cos\Theta_N|k_\alpha\rangle, \tag{S15b}$$

where the mixing angle Θ_N is

$$\Theta_N = \frac{1}{2}\tan^{-1}\left(\frac{2\sqrt{N}g_{\mathbf{k}}(k_\alpha)}{\omega_{\mathrm{ex}} + \lambda - \omega_{\mathbf{k}}(k_\alpha)}\right) \in [0, \frac{\pi}{2}). \tag{S16}$$

The dark states (Eq. S14) on the other hand, do not couple to the photonic DOF because there is no matching k index from the photonic DOF. These diabatic definitions of polariton and dark states will be used to interpret the quantum dynamics of the population relaxation process presented in Fig. 3 of the main text and in Fig. S6.

Model Parameters.

The excitonic energy was taken to be $\hbar\omega_{\mathrm{ex}} = 1.96$ eV, and the fundamental photon frequency was taken to be $\hbar\omega_{\mathrm{c}} = 1.90$ eV (which is $\omega_{\mathbf{k}}$ at the normal incidence $k_{\parallel} = 0$) for the model used in Figs. 1-3 in the main text, and $\hbar\omega_{\mathrm{c}} = 1.77$ eV in Fig. 4 (which illustrates the transition from ballistic to diffusive transport). For all simulations, $N = 10001$ molecules and $\mathcal{M} = 283$ modes were chosen, keeping the ratio of $N/\mathcal{M} \approx 35$. The inter-molecular spacing is set to be $L = 40$ Å, and the total light-matter coupling strength is set to $\sqrt{N}g_{\mathrm{c}} = 120$ meV.

II. Polariton Quantum Dynamics Propagation Method.

In this work, we treat the excitonic and photonic systems as the quantum DOF, while the phonon bath as the classical DOF. By doing this, we describe the polariton dynamics associated with $\hat{H}_{\mathrm{Q}} = \hat{H} - \hat{H}_{\mathrm{b}}$ using TDSE and evolve the phonon bath DOF associated with $\hat{H}_{\mathrm{b}} + \hat{H}_{\mathrm{ex-b}}$ using Ehrenfest mean-field force. Here, we consider the single excitation subspace in our simulation. In

this subspace, we consider the following matter or photonic excitations

$$|E_n\rangle = |\mathrm{e}_n\rangle \bigotimes_{m\neq n} |\mathrm{g}_m\rangle \bigotimes_{k_\parallel \in \{k_\alpha\}} |0_{k_\parallel}\rangle \tag{S17a}$$

$$|k_\alpha\rangle = |G\rangle \bigotimes_{k_\parallel \neq k_\alpha} |0_{k_\parallel}\rangle \otimes |1_{k_\alpha}\rangle\,, \tag{S17b}$$

where $|\mathrm{g}_n\rangle$ and $|\mathrm{e}_n\rangle$ represent the ground and excited states of the n_{th} molecule, respectively, and $|G\rangle = \bigotimes_n |g_n\rangle$ represents the matter ground state. The excitation of the α_{th} photon mode with in-plane wave-vector k_α is given by $|1_{k_\alpha}\rangle$, while $|0_{k_\alpha}\rangle$ represents the vacuum state of the α_{th} photonic mode.

Using the above bases, the quantum part of the GHTC Hamiltonian (Eq. S1) $\hat{H}_{\mathrm{Q}} = \hat{H} - \hat{H}_{\mathrm{b}}$ is expressed as

$$\hat{H}_{\mathrm{Q}} = \sum_n \hbar\omega_n\, |E_n\rangle\langle E_n| + \sum_\alpha \hbar\omega_\alpha\, |k_\alpha\rangle\langle k_\alpha| + \sum_{n\alpha} g_\alpha \left(|E_n\rangle\langle k_\alpha|\, e^{-ik_\alpha x_n} + |k_\alpha\rangle\langle E_n|\, e^{ik_\alpha x_n} \right) \tag{S18}$$

where

$$\hbar\omega_n = \hbar\omega_{\mathrm{ex}} + \lambda + \sum_\nu c_{n,\nu} R_{n,\nu}$$

is the exciton energy plus re-organization energy and the exciton-bath coupling,

$$\hbar\omega_\alpha = \hbar c\sqrt{k_\alpha^2 + k_\perp^2} \tag{S19}$$

is the cavity mode energy for mode α, and the coupling strength is

$$g_\alpha = \hbar g_{\mathrm{c}} \sqrt{\frac{\omega_{\mathbf{k}}(k_\alpha)}{\omega_{\mathbf{k}}(0)}} \cos\theta. \tag{S20}$$

A general polariton state in the single excitation basis (Eq. S17a-S17b) is expressed as

$$|\psi(t)\rangle = \underbrace{\sum_n c_n(t)\, |E_n\rangle}_{|\psi_{\mathrm{ex}}(t)\rangle} + \underbrace{\sum_\alpha c_\alpha(t)\, |k_\alpha\rangle}_{|\psi_{\mathrm{ph}}(t)\rangle} + c_0(t)|G\rangle \equiv |\psi_{\mathrm{ex}}(t)\rangle + |\psi_{\mathrm{ph}}(t)\rangle + c_0(t)|G\rangle, \tag{S21}$$

where $c_n(t)$ and $c_\alpha(t)$ are the time-dependent expansion coefficients for the excitonic and photonic DOF, $|\psi_{\text{ex}}\rangle$ and $|\psi_{\text{ph}}\rangle$ are the excitonic and photonic part of the polariton quantum state, and $c_0(t)|G\rangle$ represent the ground state contribution (due to cavity loss). The ground state part $c_0(t)|G\rangle$ will be updated from a stochastic Lindblad algorithm[8] detailed later (see Eq. S45), and not from $\hat{H}_{\text{Q}}$. Similarly, the population decay and decoherence for $c_\alpha(t)$ will also be separately updated from the stochastic Lindblad dynamics (see Eq. S44).

The polariton quantum dynamics is propagated with

$$i\hbar\frac{\partial}{\partial t}|\psi(t)\rangle = \hat{H}_{\text{Q}}(\mathbf{R})|\psi(t)\rangle, \tag{S22}$$

where $|\psi(t)\rangle$ is represented using Eq. S21. Solving Eq. S22 requires the action of Hamiltonian (Eq. S18) on the quantum state (Eq. S21), resulting in

$$\begin{aligned}
&\hat{H}_{\text{Q}}\,|\psi\rangle = \hat{H}_{\text{Q}}|\psi_{\text{ex}}\rangle + \hat{H}_{\text{Q}}|\psi_{\text{ph}}\rangle \qquad \text{(S23)}\\
&= \big(\sum_n \hbar\omega_n\,|E_n\rangle\langle E_n| + \sum_{n\alpha} g_\alpha\,|k_\alpha\rangle\langle E_n|\,e^{ik_\alpha x_n}\big)|\psi_{\text{ex}}\rangle + \big(\sum_\alpha \hbar\omega_\alpha\,|k_\alpha\rangle\langle k_\alpha| + \sum_{n\alpha} g_\alpha\,|E_n\rangle\langle k_\alpha|\,e^{-ik_\alpha x_n}\big)|\psi_{\text{ph}}\rangle\\
&= \left(\sum_n c_n\hbar\omega_n\,|E_n\rangle + \sum_{n\alpha} c_n g_\alpha e^{ik_\alpha x_n}\,|k_\alpha\rangle\right) + \left(\sum_\alpha c_\alpha\hbar\omega_\alpha\,|k_\alpha\rangle + \sum_{n\alpha} c_\alpha g_\alpha e^{-ik_\alpha x_n}\,|E_n\rangle\right),
\end{aligned}$$

where the first term in the last line of the above equation is the result of $\hat{H}_{\text{Q}}$ acting on $|\psi_{\text{ex}}\rangle$ and the second term is the result of $\hat{H}_{\text{Q}}$ acting on $|\psi_{\text{ph}}\rangle$. More specifically, for the first term, $\sum_n c_n\hbar\omega_n\,|E_n\rangle$ is the result of acting $\sum_n \hbar\omega_n\,|E_n\rangle\langle E_n|$ on $|\psi_{\text{ex}}\rangle$, and the second term is the result of acting $\sum_{n\alpha} g_\alpha\,|k_\alpha\rangle\langle E_n|\,e^{ik_\alpha x_n}$ on $|\psi_{\text{ex}}\rangle$, and similarly to the second term. We have not explicitly written the t-dependence in the above equation for conciseness.

Below, we show that Eq. S23 can be reduced into the evaluation of vector-vector Hadamard products, which have a linear scaling, and fast Fourier transforms (FFTs), which have quasi-linear scaling. The photonic subspace is defined in momentum-space (c.f. Eq. S4), with a grid spacing of

$$k_\alpha - k_{\alpha-1} = \frac{2\pi}{LN}, \tag{S24}$$

where the grid ranging from $-k_{\frac{\mathcal{M}}{2}}$ to $k_{\frac{\mathcal{M}}{2}}$. The matrix elements of the light-matter interaction

component of the Hamiltonian $\langle k_\alpha| \hat{H}_\mathrm{Q} |E_n\rangle$ is thus expressed as

$$\begin{aligned}\langle k_\alpha| \hat{H}_\mathrm{Q} |E_n\rangle &= e^{ik_\alpha x_n} g_\alpha = e^{i\left(\frac{2\pi}{LN}\right)\left(-\frac{\mathcal{M}}{2}+1+\alpha\right)(nL)} g_\alpha \\ &= e^{i\frac{2\pi n}{N}} e^{i\frac{2\pi n}{N}\left(-\frac{\mathcal{M}}{2}+\alpha\right)} g_\alpha \\ &= g_\alpha \cdot \underbrace{\left(e^{-i\frac{2\pi n}{N}\left(\frac{\mathcal{M}}{2}\right)}\right)}_{\mathcal{F}_\mathrm{shift}} \cdot \underbrace{\left(e^{i\frac{2\pi n\alpha}{N}}\right)}_{\mathcal{F}_n^{-1}} \cdot \underbrace{\left(e^{i\frac{2\pi n}{N}}\right)}_{\phi_n},\end{aligned} \tag{S25}$$

The last step can be viewed as an inverse Discrete Fourier Transform, inverse-DFT ($\mathcal{F}^{-1}$, which can be performed by inverse-FFT) of the wavefunction coefficient for n_th exciton multiplied by the phase-factor ϕ_n followed by a shift in frequency axis with the phase factor $\mathcal{F}_\mathrm{shift}$ (which can be performed by FFT shift). Note that Eq. S25 states that the contribution to the photonic component of the quantum vector (Eq. S22) due to light-matter interaction (that couples $|E_n\rangle$ with $|k_\alpha\rangle$) is the discrete Fourier transform of $|\psi_\mathrm{ex}\rangle$, which we can be efficiently evaluated with an inverse-FFT algorithm as discussed above.

Algorithm for inverse-FFT of matter component. The excitonic component of the total wavefunction only contains N coefficients corresponding to the N excitonic sites, and we need to obtain a matter wavefunction of $\mathcal{M}$ coefficients for each of the $\mathcal{M}$ photonic modes. We follow the steps below to perform Eq. S25 on the excitonic component of the wavefunction,

1. Let the photonic wavefunction be represented by the vector,

$$|\psi_\mathrm{ex}\rangle = \sum_{n=0}^{N-1} c_n |E_n\rangle \rightarrow \begin{bmatrix} c_0 \\ c_1 \\ c_2 \\ \vdots \\ c_{N-1} \end{bmatrix} \tag{S26}$$

2. Multiply the phase factor, ϕ_n in Eq. S25 with the excitonic wavefunction to obtain

$$|\psi'_{\text{ex}}\rangle \to \left[e^{i\frac{2\pi n}{N}}\right]^{\text{T}} \odot |\psi_{\text{ex}}\rangle = \begin{bmatrix} 1 \\ e^{i\frac{2\pi}{N}} \\ e^{i\frac{2\pi\cdot 2}{N}} \\ \vdots \\ e^{i\frac{2\pi\cdot(N-1)}{N}} \end{bmatrix} \odot \begin{bmatrix} c_0 \\ c_1 \\ c_2 \\ \vdots \\ c_{N-1} \end{bmatrix} = \begin{bmatrix} c_0 \\ e^{i\frac{2\pi}{N}} \cdot c_1 \\ e^{i\frac{2\pi\cdot 2}{N}} \cdot c_2 \\ \vdots \\ e^{i\frac{2\pi\cdot(N-1)}{N}} \cdot c_{N-1} \end{bmatrix}, \tag{S27}$$

where the $\odot$ represents a simple Hadamard product between the vectors.

3. Take an inverse Fast Fourier transform (iFFT) of $|\psi'_{\text{ex}}\rangle$ and perform an FFT shift on the resultant vector to obtain the photon modes in range $(-\frac{\pi}{2}, \frac{\pi}{2})$ instead of $(0, \pi)$,

$$|\psi^{\mathcal{F}}_{\text{ex}}\rangle \to \mathcal{F}_{\text{shift}}\left[\mathcal{F}^{-1}\left\{|\psi'_{\text{ex}}\rangle\right\}\right] = \mathcal{F}_{\text{shift}} \begin{bmatrix} c^{\mathcal{F}}_0 \\ c^{\mathcal{F}}_1 \\ c^{\mathcal{F}}_2 \\ \vdots \\ c^{\mathcal{F}}_{N-1} \end{bmatrix} = \begin{bmatrix} c^{\mathcal{F}}_{\frac{N}{2}} \\ c^{\mathcal{F}}_{\frac{N}{2}+1} \\ \vdots \\ c^{\mathcal{F}}_{N-1} \\ c^{\mathcal{F}}_0 \\ c^{\mathcal{F}}_1 \\ \vdots \\ c^{\mathcal{F}}_{\frac{N}{2}-1} \end{bmatrix} \tag{S28}$$

where $\mathcal{F}_{\text{shift}}$ is the action of performing a Fourier shift and can be done easily through the standard FFT libraries in Python[9] and other programming languages.

4. The photonic part of the wavefunction is obtained by selecting $\mathcal{M}$ coefficients from the center of the vector $|\psi^{\mathcal{F}}_{\text{ex}}\rangle$, and multiplying by the light-matter coupling strength g_α (as well as the number of molecules N due to the conventions of DFT as used in the NumPy library[9–11]).

$$|\psi_{\mathrm{ph}}\rangle \to N \cdot \begin{bmatrix} c^{\mathcal{F}}_{N-1-\frac{\mathcal{M}}{2}+1} \\ c^{\mathcal{F}}_{N-1-\frac{\mathcal{M}}{2}+2} \\ \vdots \\ c^{\mathcal{F}}_{N-1} \\ c^{\mathcal{F}}_{0} \\ c^{\mathcal{F}}_{1} \\ \vdots \\ c^{\mathcal{F}}_{\frac{N}{2}-1-\frac{\mathcal{M}}{2}} \end{bmatrix} \odot \begin{bmatrix} g_{-\frac{\mathcal{M}}{2}} \\ g_{-\frac{\mathcal{M}}{2}+1} \\ \vdots \\ g_{-1} \\ g_0 \\ g_1 \\ \vdots \\ g_{\frac{\mathcal{M}}{2}} \end{bmatrix}. \tag{S29}$$

The FFT Algorithm of the photonic component.

Similarly, the matrix elements of the matter-light matrix element of the Hamiltonian $\langle E_n|\hat{H}_{\mathrm{Q}}|k_\alpha\rangle$ is expressed as

$$\begin{aligned}\langle E_n|\hat{H}_{\mathrm{Q}}|k_\alpha\rangle &= e^{-ik_\alpha x_n} g_\alpha = e^{-i\frac{2\pi}{LN}x_n k_\alpha} g_\alpha = e^{-i\left(\frac{2\pi}{L}\right)\left(\frac{nL}{N}\right)\left(-\frac{\mathcal{M}}{2}+1+\alpha\right)} g_\alpha \\ &= e^{-i\left(\frac{2\pi n\alpha}{N}\right)} e^{-i\left(\frac{2\pi n}{N}\right)\left(-\frac{\mathcal{M}}{2}+1\right)} g_\alpha = e^{-i\left(\frac{2\pi n\alpha}{N}\right)} e^{-i\frac{2\pi n}{N}} e^{i\pi n\frac{\mathcal{M}}{N}} g_\alpha \\ &= \underbrace{\left(e^{-i\frac{2\pi n}{N}} e^{i\pi n\frac{\mathcal{M}}{N}}\right)}_{\phi_n} \cdot \underbrace{\left(e^{-i\left(\frac{2\pi n\alpha}{N}\right)} g_\alpha\right)}_{\mathcal{F}_\alpha},\end{aligned} \tag{S30}$$

where the phase ϕ_n depends on the excitonic site and $\mathcal{F}_\alpha$ represents the Fast Fourier Transform (FFT) coefficient for the α_{th} photonic mode.

The photonic component of the total wavefunction only contains M coefficients corresponding to the M photonic modes and we need to obtain a matter wavefunction of N coefficients for each of the N exciton sites. To obtain the FFT, we first zero-pad the photonic wavefunction to get a wavefunction of size N and then take the FFT of the bigger vector. We follow the steps below to perform Eq. S25 on the photonic component of the wavefunction,

1. Let the photonic wavefunction be represented by the vector,

$$|\psi_{\mathrm{ph}}\rangle = \sum_\alpha c_\alpha |k_\alpha\rangle \to [c_{-\frac{\mathcal{M}}{2}},\ c_{-\frac{\mathcal{M}}{2}+1},\ \ ...,\ c_{\frac{\mathcal{M}}{2}}]^{\mathrm{T}} \tag{S31}$$

2. Multiply the vector by the light-matter coupling for each mode

$$|\psi'_{\text{ph}}\rangle \to \left[g_{-\frac{\mathcal{M}}{2}} c_{-\frac{\mathcal{M}}{2}},\ g_{-\frac{\mathcal{M}}{2}+1} c_{-\frac{\mathcal{M}}{2}+1},\ \ ...,\ g_{\frac{\mathcal{M}}{2}} c_{\frac{\mathcal{M}}{2}}\right]^{\text{T}} \tag{S32}$$

3. Zero pad the above vector to get the vector to the same dimensionality as the exciton coefficients

$$|\psi^{N}_{\text{ph}}\rangle \to \Big[\underbrace{g_{-\frac{\mathcal{M}}{2}} c_{-\frac{\mathcal{M}}{2}},\ g_{-\frac{\mathcal{M}}{2}+1} c_{-\frac{\mathcal{M}}{2}+1},\ \ ...,\ g_{\frac{\mathcal{M}}{2}} c_{\frac{\mathcal{M}}{2}},}_{\mathcal{M}}\ \ \underbrace{0,\ \ 0,\ \ ...,\ \ 0}_{N-\mathcal{M}}\Big]^{\text{T}} \tag{S33}$$

4. Take a Fast Fourier Transform of the above vector

$$\mathcal{F}\left\{|\psi^{N}_{\text{ph}}\rangle\right\} \to \mathcal{F}\left\{\Big[\underbrace{g_{-\frac{\mathcal{M}}{2}} c_{-\frac{\mathcal{M}}{2}},\ g_{-\frac{\mathcal{M}}{2}+1} c_{-\frac{\mathcal{M}}{2}+1},\ \ ...,\ g_{\frac{\mathcal{M}}{2}} c_{\frac{\mathcal{M}}{2}},}_{\mathcal{M}}\ \ \underbrace{0,\ \ 0,\ \ ...,\ \ 0}_{N-\mathcal{M}}\Big]^{\text{T}}\right\} \tag{S34}$$

5. To obtain the matter wavefunction, multiply the above vector with the phase factor in Eq. S30

$$|\psi_{\text{ex}}\rangle = \left[e^{-i\frac{2\pi n}{N}} e^{\frac{i\pi n M}{N}}\right]^{\text{T}} \odot \mathcal{F}\left\{|\psi^{N}_{\text{ph}}\rangle\right\} = \begin{bmatrix} 1 \\ e^{-i\frac{2\pi}{N}} e^{\frac{i\pi\mathcal{M}}{N}} \\ e^{-i\frac{2\pi\cdot 2}{N}} e^{\frac{i\pi\cdot 2\mathcal{M}}{N}} \\ \vdots \\ e^{-i\frac{2\pi\cdot(\mathcal{M}-1)}{N}} e^{\frac{i\pi\cdot(\mathcal{M}-1)\mathcal{M}}{N}} \\ e^{-i\frac{2\pi\cdot\mathcal{M}}{N}} e^{\frac{i\pi\cdot\mathcal{M}^2}{N}} \\ e^{-i\frac{2\pi\cdot(\mathcal{M}+1)}{N}} e^{\frac{i\pi\cdot(\mathcal{M}+1)M}{N}} \\ \vdots \\ e^{-i\frac{2\pi\cdot(N-1)}{N}} e^{\frac{i\pi\cdot(N-1)\mathcal{M}}{N}} \end{bmatrix} \odot \mathcal{F} \begin{bmatrix} g_{-\frac{\mathcal{M}}{2}} c_{-\frac{\mathcal{M}}{2}} \\ g_{-\frac{\mathcal{M}}{2}+1} c_{-\frac{\mathcal{M}}{2}+1} \\ g_{-\frac{\mathcal{M}}{2}+2} c_{-\frac{\mathcal{M}}{2}+2} \\ \vdots \\ g_{\frac{\mathcal{M}}{2}} c_{\frac{\mathcal{M}}{2}} \\ 0 \\ 0 \\ \vdots \\ 0 \end{bmatrix} \tag{S35}$$

Consequently, Eqn. S30 shows that the contribution to the matter component of the wavefunction due to matter-light interaction is the discrete Fourier transform of $\tilde{g}_\alpha \cdot c_\alpha$, which we can evaluate with an inverse FFT. From Eqn. S25 and Eqn. S30, we re-express the action of GHTC-Hamiltonian on the wavefunction in Eq. S23 as,

$$\hat{H}_{\text{Q}}\,|\psi\rangle = |\epsilon_\psi\rangle \odot |\psi\rangle + \left(\mathcal{F}^{-1}\,|\psi_{\text{ex}}\rangle \oplus \mathcal{F}\,|\psi_{\text{ph}}\rangle\right) \tag{S36}$$

where $\mathcal{F}$ and $\mathcal{F}^{-1}$ refer to the fast Fourier and inverse Fourier transforms respectively and the $|\epsilon_\psi$ is the vector containing the energies of the exciton sites (ϵ_{E_n} = energy of the n_{th} exciton site) and the photonic modes (ϵ_{k_α} = energy of the α_{th} photonic mode),

$$|\epsilon_\psi\rangle \rightarrow \Big[\underbrace{\epsilon_{E_0},\ \epsilon_{E_1},\ \epsilon_{E_2},\ \ \ldots,\ \epsilon_{E_{N-1}}}_{\text{Exciton site energies}},\ \underbrace{\epsilon_{k_{-\mathcal{M}/2}},\ \ \ldots, \epsilon_{k_0}, \ldots\ \epsilon_{k_{\mathcal{M}/2}}}_{\text{Photon mode energies}}\Big]^{\text{T}} \tag{S37}$$

where the site energy ϵ_{E_i} fluctuates around the mean exciton energy $\hbar\omega_{\text{ex}}$ (Eq. S5), as given by Eq. S9,

$$\epsilon_{E_n} = \hbar\omega_{\text{ex}} + \lambda + \hbar\sum_\nu c_{n,\nu}R_{n,\nu} \tag{S38}$$

and the energy of the α_{th} is given by Eq. S3 and Eq. S4,

$$\epsilon_{k_\alpha} = \hbar c\sqrt{\left(\frac{2\pi}{NL}\alpha\right)^2 + k_\perp^2}. \tag{S39}$$

Eq. S36 shows that the action of the GHTC Hamiltonian is reduced into a vector-vector Hadamard product[12] and two FFT operations, which significantly speeds up the computations as the vectorized Hadamard operation scale as $\mathcal{O}(N)$ and the FFT operation scale as $\mathcal{O}(N\log(N))$. Moreover, by recasting the action of the Hamiltonian into an FFT, we are able to efficiently calculate the Hamiltonian acting on a quantum state vector, without generating the $\mathcal{O}(N^2M^2)$ size matrix. This reduces the memory requirement for the dynamics of large molecular systems, where N is around $10^4 - 10^6$.

The quasi-linear scaling relation is illustrated in Fig. S2, where we plot the run-time of one $\hat{H}_{\text{Q}}|\psi\rangle$ operation against the size of the $\hat{H}_{\text{Q}}$ matrix in a log-log scale. Here, we report the operation time for normal matrix-vector multiplication (MVM) $\hat{H}_{\text{Q}}|\psi\rangle$ (normal MVM, blue color), MVM that takes advantage of sparsity only in $\hat{H}_{\text{Q}}$ (sparse MVM, red), and MVM that take advantage of both sparsity and the FFT algorithm (FFT MVM, green). We note that the run-time we evaluated for the FFT MVM in Fig. S2 includes additional overhead for calling the FFT routine, which partially accounts for the increased run-time observed for small matrix sizes in comparison to the other MVM methods. Further, we chose matrix sizes in powers of 2 to take advantage of efficient FFT algorithms implemented for radix-2.[10]

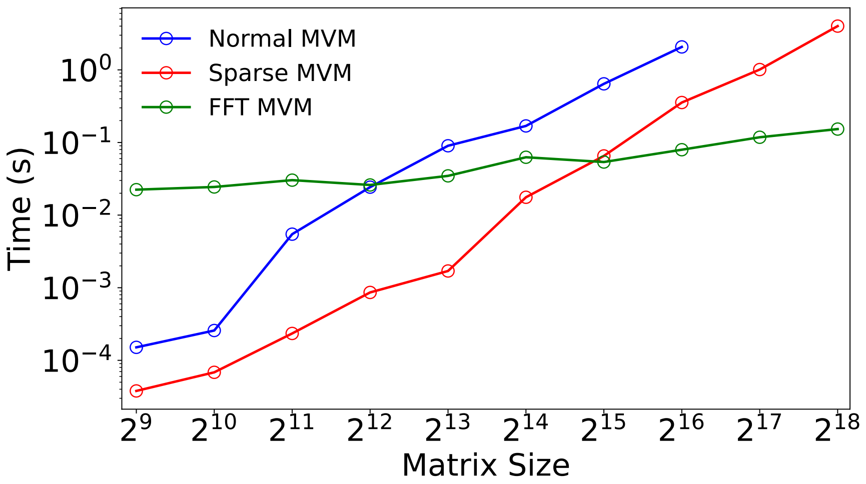

Figure S2: Run-times of normal MVM (blue), sparse MVM (red), and FFT MVM (green) as a function of matrix size for the MVM operation $\hat{H}_\mathrm{Q}|\psi\rangle$.

Fig. S2 shows that for smaller matrices (matrix size $\sim 2^9 - 2^{12}$), the sparse MVM is faster than the normal MVM and the FFT MVM. We anticipate that the FFT MVM will be as fast as the sparse MVM within this range once the overhead of the FFT is accounted for. As the matrix size increases, the run-time of the normal MVM surpasses that of the FFT MVM when the matrix reaches a size of 2^{12}, while the run-time of the sparse MVM exceeds the run-time of the FFT MVM at a matrix of size 2^{15}. This is due to the unfavorable computational scaling of the normal and sparse MVMs, which is $\mathcal{O}\big(N^2\big)$, in contrast to the FFT MVM, which is $\mathcal{O}(N\log(N))$. Due to the quasi-linear $\mathcal{O}(N\log(N))$ scaling of the FFT MVM, the run-time exhibits a nearly flat dependence on matrix size for the range of matrix sizes considered. We further note that our computational resources were insufficient to manage the memory requirements of the normal MVM for matrices exceeding size 2^{16} and thus, we do not report run-times for normal MVM beyond matrix sizes of 2^{16}. The FFT algorithm, on the other hand, does not have such a restriction.

Details of the $\mathcal{L}$-MFE approach.

To perform the transport simulations, Ehrenfest dynamics is employed, where we simulate an ensemble of trajectories with varied initial conditions. Within each trajectory, the exciton-photon wavefunction is propagated in accordance with the equation

$$-i\hbar\dot{\mathbf{c}}(t) = \hat{H}_\mathrm{Q}(\mathbf{R}(t))\mathbf{c}(t), \tag{S40}$$

where $\mathbf{c}(t) = [\{c_n(t)\}, \{c_\alpha(t)\}]^{\mathrm{T}}$ are the expansion coefficients, with a total of N exciton coefficients $c_n(t)$, a total of $\mathcal{M}$ photonic coefficient $c_\alpha(t)$. The nuclear coordinates are updated according to Newton's equation of motion

$$\frac{\partial R_{n,\nu}}{\partial t} = P_{n,\nu}, \quad \frac{\partial P_{n,\nu}}{\partial t} = \mathcal{F}_{n,\nu} \tag{S41}$$

where the bath modes experience the force from the potential energy surface generated by $|\psi(t)\rangle$,

$$\begin{aligned}\mathcal{F}_{n,\nu} &= -\nabla_{R_{n,\nu}} \langle \psi(t)|(\hat{H}_{\mathrm{ex-b}} + \hat{H}_{\mathrm{b}})|\psi(t)\rangle = -|c_n(t)|^2 \cdot \langle E_n|\nabla_{R_{n,\nu}} \hat{H}|E_n\rangle, \\ &= -|c_n(t)|^2 \cdot (C_{n,\nu} + \omega_\nu R_{n,\nu}),\end{aligned} \tag{S42}$$

where we used the diagonal structure of bi-linear system-bath interaction in the HTC Hamiltonian (Eq. S9), and only $|E_n\rangle$ has the exciton-phonon coupling (whereas $|k_\alpha\rangle$ does not). Here, $C_{n,\nu}$ is the exciton-phonon coupling strength between phonon mode $R_{n,\nu}$ and exciton state $|E_n\rangle$, see Eq. S9.

The cavity loss (photon loss) dynamics from state $|k_\alpha\rangle$ to state $|G\rangle$ is formally described using the Lindblad super operator $\hat{L}_\alpha = |G\rangle\langle k_\alpha|$. The dissipator $\mathcal{L}$ accounts for the cavity loss channel causing the system to relax

$$\mathcal{L}[\hat{\rho}_{\mathrm{Q}}] = \sum_\alpha \Gamma_{\mathrm{c}} \Big(\hat{L}_\alpha \hat{\rho}_{\mathrm{Q}} \hat{L}_\alpha^\dagger - \frac{1}{2}\{\hat{L}_\alpha^\dagger \hat{L}_\alpha, \hat{\rho}_{\mathrm{Q}}\} \Big), \tag{S43}$$

which can be formally unravelled[8] as

$$c_\alpha(t + dt) = c_\alpha(t) e^{-\Gamma_{\mathrm{c}} dt}, \tag{S44}$$

and the coefficients of the ground state change by the amount

$$c_0(t + dt) = e^{i\phi} \sqrt{|c_0(t)|^2 + (1 - e^{-\Gamma_\alpha dt})|c_\alpha(t)|^2}, \tag{S45}$$

where the random phase ϕ is drawn from a uniform distribution

$$\mathcal{P}(\phi) = \frac{1}{2\Delta_\phi} \quad , \quad \Delta_\phi \le \phi \le \Delta_\phi \tag{S46}$$

and the width of this distribution is determined by numerically solving the transcendental equation

$$\frac{\sin(\Delta_\phi)}{\Delta_\phi} = \frac{|c_0(t)|}{\sqrt{|c_0(t)|^2 + (1 - e^{\Gamma_c dt})|c_\alpha(t)|^2}}. \tag{S47}$$

Details of this algorithm can be found in Ref. 8.

III. Details of Quantum Dynamics Simulations.

Polaritonic Initial Conditions.

We used polariton wavepackets that are localized over the molecules but centered around a given value of k_α as the initial conditions. To generate these wavepackets, we define the polariton states $|\Phi_J\rangle$ as follows

$$\hat{H}_{\rm pl} |\Phi_J\rangle = \mathcal{E}_J |\Phi_J\rangle \,, \tag{S48}$$

where $\hat{H}_{\rm pl} = \hat{H}_{\rm ex} + \hat{H}_{\rm ph} + \hat{H}_{\rm LM}$, and the state $|\Phi_J\rangle$ has the polariton energy $\mathcal{E}_J$. Additionally, we note that the polariton states obtained from diagonalization take the form

$$|\Phi_J\rangle = \sum_n \tilde{c}_{n,J} |E_n\rangle + \sum_\alpha \tilde{c}_{\alpha,J} |k_\alpha\rangle \,, \tag{S49}$$

where $\tilde{c}_{n,J}$ and $\tilde{c}_{\alpha,J}$ are the excitonic and photonic components of the $J_{\rm th}$ polariton state, respectively.

To obtain a spatially localized polaritonic initial state, corresponding to a specific energy window $\mathcal{E} \in [\mathcal{E}_0 - \Delta\mathcal{E}/2, \mathcal{E} + \Delta\mathcal{E}/2]$ (generated from initial laser excitation), we expand the initial state in terms of the polariton states defined in Eq. (S48) as follows

$$|\psi(0)\rangle = \sum_J a_J |\Phi_J\rangle, \tag{S50}$$

where $a_J = \langle\Phi_J|\psi(0)\rangle$ is the expansion coefficient of the initial state $|\psi(0)\rangle$ onto $|\Phi_J\rangle$, with a_J yet to be determined. For the initial excitation in the energy window $\mathcal{E} \in [\mathcal{E}_0 - \Delta\mathcal{E}/2, \mathcal{E} + \Delta\mathcal{E}/2]$, we consider those $\{|\Phi_J\rangle\}$ such that the polariton energy $\mathcal{E}_J \in [\mathcal{E}_0 - \Delta\mathcal{E}/2, \mathcal{E} + \Delta\mathcal{E}/2]$. The coefficients a_J are then generated by minimizing the spread of the wave packet Δx^2 in the excitonic space,

defined as[3]

$$\Delta x^2 = \sum_n \rho_n x_n^2 - \sum_n (\rho_n x_n)^2, \tag{S51}$$

where $\rho_n = |\langle E_n|\psi(0)\rangle|^2 / \sum_m |\langle E_m|\psi(0)\rangle|^2$ is the normalized probability of occupying molecule located at x_n, and $|E_n\rangle$ is defined in Eq. S17a. In the single-excited diabatic basis, ρ_n is expressed as

$$\rho_n = \frac{|\sum_J a_J \tilde{c}_{n,J}|^2}{\sum_m |\sum_J a_J \tilde{c}_{m,J}|^2}, \tag{S52}$$

and the spread of the wavepacket Δx^2 is given by

$$\Delta x^2 = \sum_n \frac{x_n^2 |\sum_J a_J \tilde{c}_{n,J}|^2}{\sum_m |\sum_J a_J \tilde{c}_{m,J}|^2} - \left(\frac{x_n |\sum_J a_J \tilde{c}_{n,J}|^2}{\sum_m |\sum_J a_J \tilde{c}_{m,J}|^2} \right)^2. \tag{S53}$$

Eq. (S53) is used as inputs in the basin hopping algorithm to determine the optimal set of coefficients $\{a_J\}$ that minimizes the spread of the wavepacket. Here, we use the basin hopping algorithm[13] to determine an optimal set $\{a_J\}$ that satisfies the above condition. To streamline the computations, we note that the number of polariton states within a given energy window is significantly smaller than the total number of states in the system. Thus, we use an iterative eigenvalue algorithm, specifically the ARPACK library,[14] to isolate the relevant polariton states for the optimization task.

Nuclear Initial Conditions.

Initial bath conditions sampled from the analytic form of the Wigner distribution for a Gaussian bath

$$[\hat{\rho}_{\mathrm{R}}]_{\mathrm{W}}(\mathbf{R},\mathbf{P}) = \prod_{n,\nu} 2\tanh\left(\frac{\beta\hbar\omega_\nu}{2}\right) \exp\left\{ -\tanh\left(\frac{\beta\hbar\omega_\nu}{2}\right) \left(\frac{\omega_\nu^2 R_{n,\nu}^2}{\hbar^2} + \frac{P_{n,\nu}^2}{\hbar^2\omega_\nu^2} \right) \right\} \tag{S54}$$

which are sampled using the Gaussian random number generator based on the Box–Muller method.

Simulation Details.

All results are obtained with an ensemble of 250 independent trajectories. Convergence tests are performed with up to 1000 trajectories. The nuclear time step used in $\mathcal{L}$-MFE method is $\Delta t = 2.5$ fs, where during each nuclear propagation, there are 100 electronic propagation steps with a time step $dt = 0.025$ fs. The nuclear EOM in Eq. S41 is numerically integrated with the velocity verlet

algorithm (with Force expression in Eq. S41) and the TDSE (Eq. S22) is solved with the RK4 algorithm by taking advantage of the FFT algorithm in Eq. S36. For each electronic time step, the Stochastic Lindblad loss described in Eq. S44-Eq. S45 is applied to update the photonic coefficients in order to capture the Lindblad loss dynamics of the cavity modes.

IV. Details of Polariton Transport Properties Calculations

Spacial Distribution of the Polariton Wavepacket.

To interpret the spatial distribution of the polariton wavepacket at time t and position x_n, we define the time-dependent polariton state (without the ground state component)

$$|\psi(t)\rangle = \sum_{n=0}^{N-1} c_n(t)\,|E_n\rangle + \sum_{\alpha} c_\alpha(t)\,|k_\alpha\rangle \equiv |\psi_{\rm ex}(t)\rangle + |\psi_{\rm ph}(t)\rangle, \tag{S55}$$

and define the spatial distribution due to the excitonic part $|\psi_{\rm ex}(t)\rangle$ and photonic part $|\psi_{\rm ph}(t)\rangle$ separately. The polariton states $|\pm, n\rangle$ in real space are obtained by taking a discrete Fourier transform of Eqs. S15a and S15b using the basis $e^{ik_\alpha x_n} = e^{i\frac{2\pi\alpha}{NL}\cdot(nL)} = e^{i\frac{2\pi n\alpha}{N}}$.

This leads to the following expressions

$$|+, n\rangle = \sum_{\alpha}\left[\sum_{n'=0}^{N-1}\frac{X_{k_\alpha}}{N}e^{ik_\alpha(x_n - x_{n'})}|E_{n'}\rangle + C_{k_\alpha}\frac{e^{ik_\alpha x_n}}{\sqrt{N}}|k_\alpha\rangle\right], \tag{S56a}$$

$$|-, n\rangle = -\sum_{\alpha}\left[\sum_{n'=0}^{N-1}\frac{C_{k_\alpha}}{N}e^{ik_\alpha(x_n - x_{n'})}|E_{n'}\rangle - X_{k_\alpha}\frac{e^{ik_\alpha x_n}}{\sqrt{N}}|k_\alpha\rangle\right], \tag{S56b}$$

where $C_{k_\alpha} = \sin\Theta_N$ and $X_{k_\alpha} = \cos\Theta_N$ are the Hopfield coefficients of the polariton state with wavevector k_α. The spatial distribution of the polariton wavepacket is thus given by $|\psi_\pm(x_n, t)|^2 =$

$|\langle \pm, n|\psi(t)\rangle|^2$, and the full expression for $|\psi_\pm(x_n,t)|^2$ is

$$\begin{aligned}|\psi_+(x_n,t)|^2 &= \left|\sum_\alpha \sum_{n'=0}^{N-1} \frac{X_{k_\alpha}}{N} e^{ik_\alpha(x_n - x_{n'})} c'_n\right|^2 + \left|\sum_\alpha C_{k_\alpha} \frac{e^{ik_\alpha x_n}}{\sqrt{N}} c_\alpha\right|^2 \\ &+ 2\mathrm{Re}\left[\sum_\alpha \sum_{n'=0}^{N-1} \frac{X_{k_\alpha}}{N} e^{ik_\alpha(x_n - x_{n'})} c'_n \times \left(\sum_{\alpha'} C_{k_{\alpha'}} \frac{e^{ik_{\alpha'} x_n}}{\sqrt{N}} c_{\alpha'}\right)^*\right], \end{aligned} \tag{S57a}$$

$$\begin{aligned}|\psi_-(x_n,t)|^2 &= \left|\sum_\alpha \sum_{n'=0}^{N-1} \frac{C_{k_\alpha}}{N} e^{ik_\alpha(x_n - x_{n'})} c'_n\right|^2 + \left|X_{k_\alpha} \frac{e^{ik_\alpha x_n}}{\sqrt{N}} c_\alpha\right|^2 \\ &- 2\mathrm{Re}\left[\sum_\alpha \sum_{n'=0}^{N-1} \frac{C_{k_\alpha}}{N} e^{ik_\alpha(x_n - x_{n'})} c'_n \times \left(\sum_{\alpha'} X_{k_{\alpha'}} \frac{e^{ik_{\alpha'} x_n}}{\sqrt{N}} c_{\alpha'}\right)^*\right]. \end{aligned} \tag{S57b}$$

The last term in Eqs. S57a and S57b describe the interference between the excitonic part and the photonic part of the polariton wavepacket. These *interference* terms between the exciton wavepacket and photonic wavepacket, which were included in the Gross–Pitaevskii equation,[15] were omitted in the previous work using Enrenfest dynamics simulations.[4] We found that the interference terms have a non-negligible contribution in computing polariton MSDs and group velocities.

The dark state wavepacket in real space is obtained by taking the discrete Fourier transform of Eq. S14, and this gives

$$|\mathrm{D}, n\rangle = \sum_\kappa \frac{1}{N} \sum_{n'=0}^{N-1} e^{ik_\kappa(x_n - x_{n'})} |E_{n'}\rangle. \tag{S58}$$

The spatial distribution of the dark state wavepacket is given by $|\psi_\mathrm{D}(x_n,t)|^2 = |\langle \mathrm{D}, n|\psi(t)\rangle|^2$, and is computed as

$$|\psi_\mathrm{D}(x_n,t)|^2 = \left|\sum_\kappa \frac{1}{N} \sum_{n'=0}^{N-1} e^{ik_\kappa(x_n - x_{n'})} c_{n'}\right|^2 \tag{S59}$$

We compute the polariton group velocities and MSDs from the contributions of the polariton and dark state wavepacket, that is, $|\psi(x_n,t)|^2 = |\psi_\pm(x_n,t)|^2 + |\psi_\mathrm{D}(x_n,t)|^2$.

In Fig. S3, we directly compare the transient MSD results with the interference term (solid lines) and without the interference term (dashed line). One can see that for a broadband UP excitation in panel (a), the interference term is constructive, that is, including it provides a larger value of MSD. This is because, for the UP states, the expansion coefficients for the photonic and for the excitonic component in Eq. S15 have the same sign. For a broadband LP excitation in panel (b), the interference effects lead to a smaller MSD, and the interference term provides a destructive

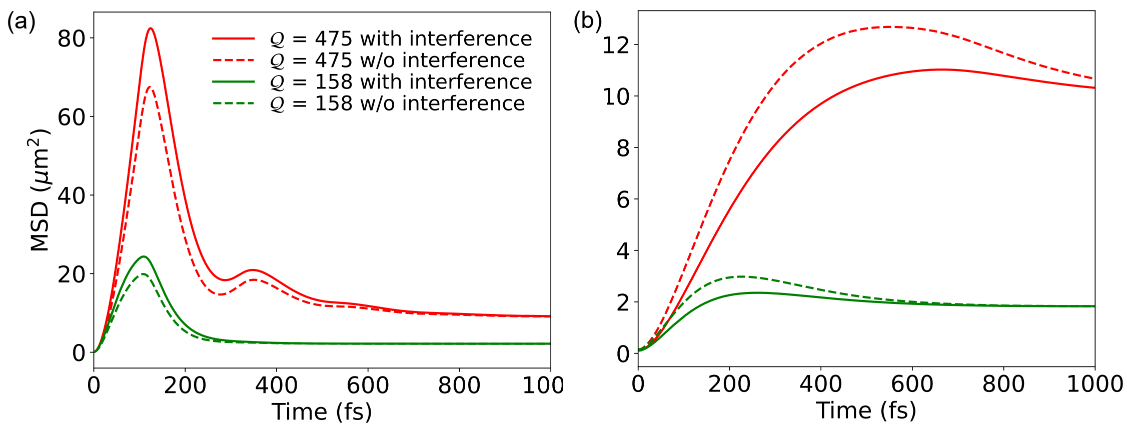

Figure S3: Comparison of the transient MSD with the interference term (solid line) and without the interference term (dashed lines), for (**a**) initial UP board band excitation (same as Fig. 2 of the main text) and (**b**) initial LP board band excitation (same as Fig. 3 of the main text).

interference effect. For the LP states, the expansion coefficients for the photonic and for the excitonic component in Eq. S15 have the opposite sign. More specifically, for the particular k_α that gives rise to zero light-matter detuning (such that $\omega_{\mathrm{ex}} + \lambda = \omega_{\mathbf{k}}$), we have $|\pm\rangle = \frac{1}{\sqrt{2}}[|k_\alpha\rangle \pm |\mathrm{B}_\alpha\rangle]$. Thus, for initial excitation in the UP branch, the interference term is constructive and for initial excitation in the LP branch, the interference is destructive.

Polariton Group Velocities.

We adopt the approach described in Ref. 3 to compute the group velocities of the polariton propagation. The group velocities are determined by tracking the wavefront of the polariton wavepacket obtained from quantum dynamics simulations. To assess the wavefront of a wavepacket, we calculate the value $\tilde{N}(t)$ such that the cumulative normalized probability of occupying molecule $n \in [0, \tilde{N}]$ (sites start from the left boundary of the simulation box till the wavefront) $P_{\mathrm{t}}(t)$, defined as

$$P_{\mathrm{t}}(t) = \sum_{n=0}^{\tilde{N}(t)} \rho_n(t) \geq 0.03. \tag{S60}$$

meets a specified threshold mentioned above, which we use the same as reported in Ref. 3. In the above expression, ρ_n is the normalized probability density of the polariton wavepacket for position x_n at time t, expressed as

$$\rho_n = \frac{|\psi(x_n, t)|^2}{\sum_m |\psi(x_m, t)|^2} \tag{S61}$$

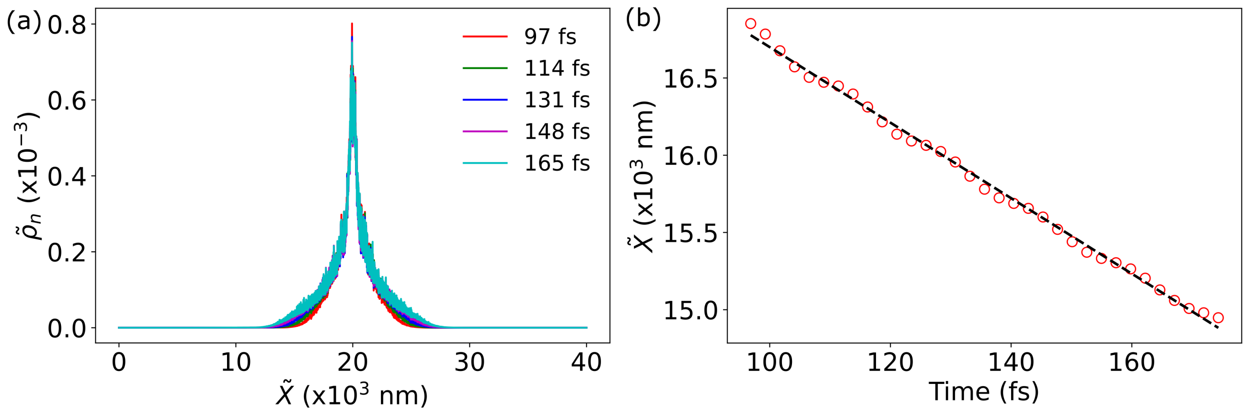

Figure S4: (**a**) Spatial distribution of polariton wavepacket at different times and (**b**) group velocities v_g extracted by tracking the wavefront of the polariton wavepacket and doing a linear fitting.

The threshold P_t was chosen to ensure that we are tracking the the wavefront, which is approximately two standard deviations from the center location of the initial wavepacket. We monitor the wavefront of the polaritonic wavepacket by tracking $\tilde{N}(t)$ and determining the position of the trailing edge $\tilde{X}(t) = \tilde{N}(t) \cdot L$. The resultant velocities v_g are derived by linearly fitting the slope of $\tilde{X}(t)$ over time. This involves computing $\mathrm{d}\tilde{X}(t)/\mathrm{d}t$, with a fitting range chosen between $t = 98$ fs and $t = 175$ fs. Fig. S4 presents one example of (a) the $\rho_n(x_n, t)$ for various propagation times, and (b) the fitting to extract v_g for broadband UP excitation in a cavity with $\mathcal{Q} = 475$, and we used the same fitting parameters ($P_\mathrm{t}(t) \geq 0.03$ and fitting time range between $t = 98$ fs and $t = 175$ fs) for all velocity reported in Figs. 1-3 in the main text.

V. Additional Numerical Results

Broad band excitation on LP branch.

Fig. S5a presents a similar study of how $\mathcal{Q}$-factor impacts v_g of polaritons, but with a broadband excitation on the LP branch (see gray Gaussian initial excitation on the LP branch in the inset). As demonstrated in Fig. S5, v_g increases with increasing $\mathcal{Q}$, reaching a maximum value of about 35 μm/ps as $\mathcal{Q} \to \infty$. We note that the maximum attainable velocity for an LP wavepacket is lower than that for an UP wavepacket. This is largely due to the curvature of the polariton dispersion curve, where steeper slopes for UP lead to larger group velocities.

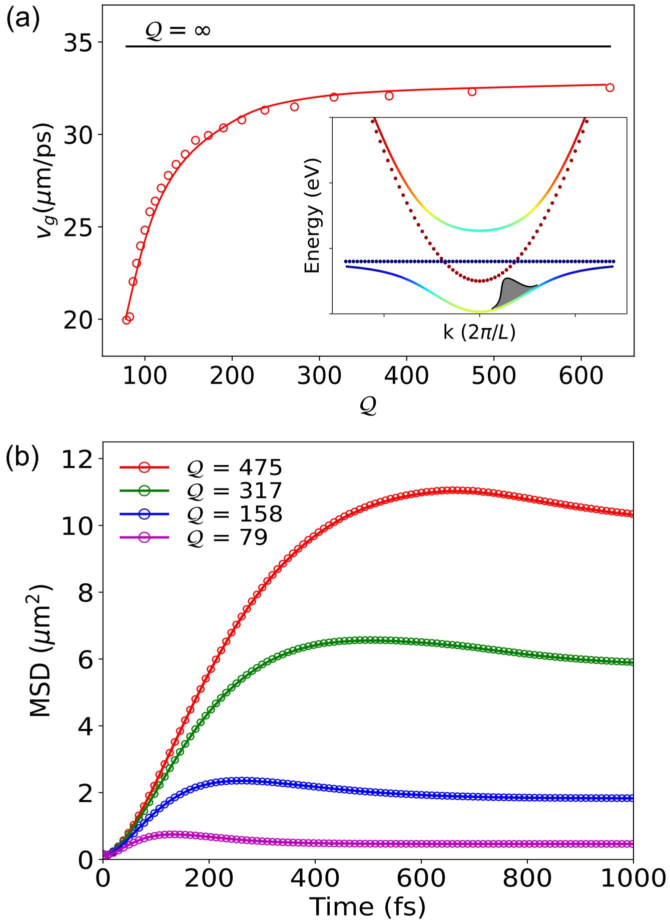

Figure S5: **Group velocity dependence on quality-factor for LP broadband excitation.** (**a**) Polariton group velocity v_g vs cavity quality factor $\mathcal{Q}$. The inset figure illustrates the energy bandwidth used for the initial excitation while maximizing the localization of the polariton wavepacket. (**b**) Time-dependent transient MSD with LP initial excitation for various $\mathcal{Q}$.

Fig. S5b presents the transient MSD computed from Eq. S64 with the LP initial excitation. In contrast to UP initial excitation (see Fig. 2b in the main text), the transient MSDs for an LP wavepacket excitation exhibit an increase followed by stabilization over time, without further collapse of the polariton wavepacket. Increasing the $\mathcal{Q}$-factor results in corresponding increases in both the wavepacket's steady-state MSD and the associated rising time. To the best of our knowledge, there are no direct experimental measurements to verify the behavior of Fig. S5b, and the same experimental setup in Ref. 16 could be used to verify this behavior once the initial excitation is set to be on LP.

Polariton Quantum Dynamics for $\mathcal{Q}$=79 system.

Here, we present the same type of data analysis as we did in Fig. 4 of the main text, for a low Q cavity with $Q = 79$. Fig. S6a and Fig. S6b depict the populations of the polaritonic states under a broadband UP and LP excitation, respectively, with cavity $\mathcal{Q}$-factors of 79 respectively. Compared to the high $\mathcal{Q}$ case presented in Fig. 4 of the main text, one can observe a more pronounced ground state population rise, due to the faster cavity loss process (with a lower $\mathcal{Q}$). Compared to the high $\mathcal{Q}$ case in Fig. 4 of the main text, here, with UP excitation, there is more population directly transferred to the ground state than the dark states, again, due to the large cavity loss rate.

For a lossy cavity with the LP initial excitation shown here (Fig. S6b), the decrease in the LP population is more rapid compared to the high $\mathcal{Q}$ cavity case in Fig. 4b of the main text, and the rate of population transfer to the ground state is greater than the rate of population transfer to the dark states. Additionally, it is observed that in the high $\mathcal{Q}$ cavity (Fig. 4 of the main text), the steady-state population of dark states is larger than the steady-state population of dark states in the lossy cavity. This is because, in the high $\mathcal{Q}$ cavity, the rate of transfer from the LP state to the dark states is greater than the rate of transfer from the LP state to the ground state, causing more population to accumulate in the dark states. Once the excitation is stored in the dark state, it is safe from cavity loss as it contains very little photonic component. In both scenarios, the populations of the UP are negligible, which agrees with previous findings that transitions from LP to UP state, and from dark state to UP state occur on much longer timescales.[17,18]

As we illustrated for broadband UP excitation in the main text, a deeper understanding of the transient MSD for broadband LP excitation is achieved by examining the polariton wavepacket population density in position space, as shown in Fig. S6c and Fig. S6d. The initial LP wavepacket is also centered at $x_0 = 20$ μm with $x \in [0, 40]\mu$m. Over time, the LP wavepacket propagates outward from the center as seen from the LP population density (12 fs to 212 fs). Due to exciton-phonon coupling, the LP wavepacket transfers the population to the dark state, increasing the dark-state population. After $\sim$ 212 fs, the dark-state wavepacket adopts a shape corresponding to the cumulative motion of the LP wavepacket. Moreover, the dark-state wavepacket remains immobile, as it primarily consists of excitons. Within a lossy cavity ($\mathcal{Q} = 79$), the LP wavepacket does not spread as much as it does in a high $\mathcal{Q}$ cavity. Thus, the dark-state wavepacket is narrower than in the high $\mathcal{Q}$ cavity, an observation that accounts for the reduced MSD in the lossy cavity

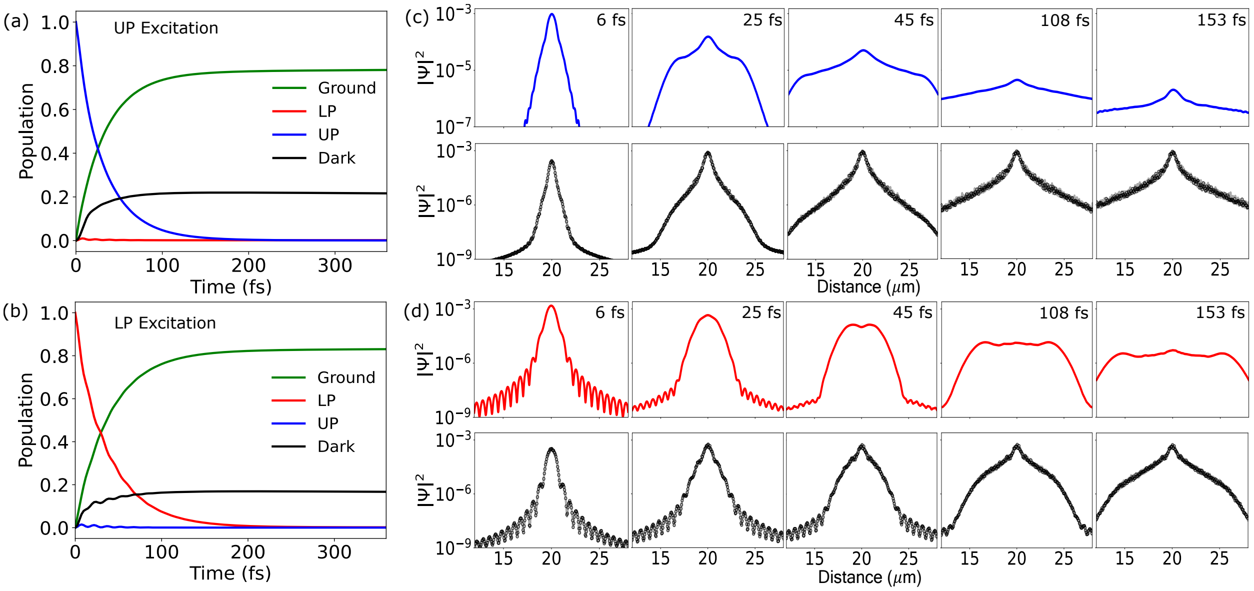

Figure S6: The populations of UP (blue), LP (red), dark (black), and ground states (green) in a lossy cavity ($\mathcal{Q} = 79$) are presented, with (**a**) broadband UP excitation and (**b**) broadband LP excitation. The wavepackets in position space, decomposed into polariton and dark state components, are illustrated for (**c**) broadband UP excitation and for (**d**) broadband LP excitation.

compared to the high $\mathcal{Q}$ cavity. Additionally, we note that the LP wavepacket spreads out less compared to the UP wavepacket (see Fig. 4c and Fig. 4d in the main text) because of the lower LP wavepacket group velocities (from the derivative of the dispersions). This results in a smaller MSD as depicted in the transient MSD plots, where the LP transient MSD lacks a pronounced spike before reaching a steady state.

Extracting v_g from transient MSD.

Note that experimentally,[16] v_g was extracted from the transient MSD. We follow the previous theoretical work[4] and further account the transient differential transmission signal in MSD measurement and use it to extract v_g. This also generates $\mathcal{Q}$-dependent v_g, consistent with the experiments[16] and the early theoretical work.[4] On the other hand, the v_g reported from Ref. 16 and extracted from $\sigma^2(t)$ represents a more "global" measure of the overall polariton wavepacket motion, and the v_g reported in Ref. 3 or extracted is related to the wavefront of the polariton.

We follow the procedure in Ref. 16 to extract the polariton group velocities v_g from the transient MSD. For ballistic propagation, the transient MSD at time t is proportional to v_g^2 and is proposed

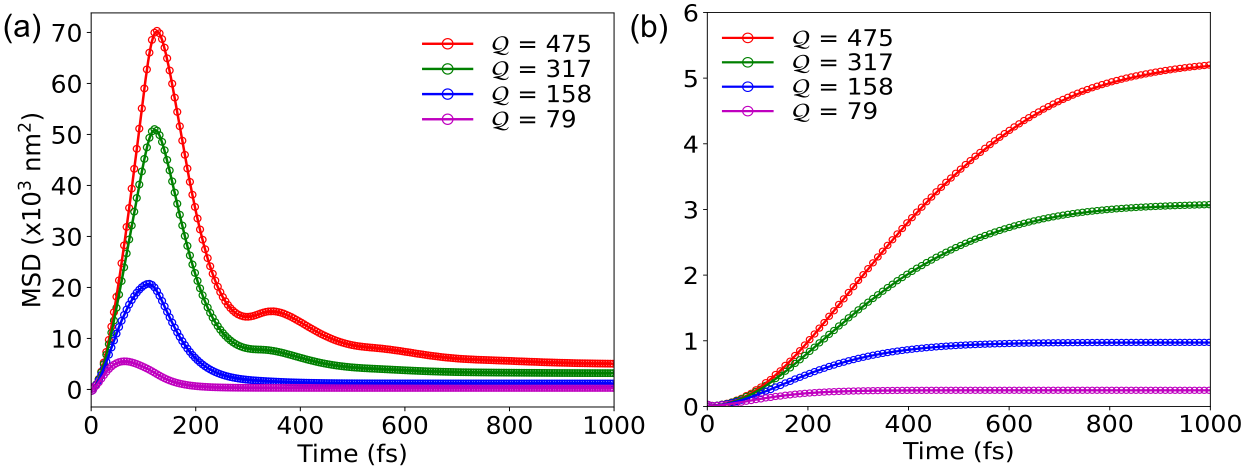

Figure S7: Transient MSD computed from Eq. S63 using the transient differential transmission signal for a (**a**) broadband UP wavepacket excitation and (**b**) broadband LP wavepacket excitation.

to have the following expression[16]

$$\sigma^2(t) = \sigma_0^2 + e^{-t/\tau} \cdot \frac{v_g^2 t^2}{\sigma_0^2}, \tag{S62}$$

where σ_0 is the MSD at a time t_0 where there is no time delay between the pump and probe signal, and τ is the coherence time of the wavepacket. To model the signal that is consistent with the experimental measurements reported in Ref. 16 (where the authors are measuring transient absorption), we scale our theoretical MSD (defined in Eq. 9 of the main text) based on the following expression suggested in Ref. 4

$$\sigma^2(t) = \sum_{n=0}^{N-1} \left(e^{\eta d |\psi(x_n,t)|^2} - 1 \right) \cdot (x_n - \langle x \rangle)^2, \tag{S63}$$

where η is the absorption coefficient of the sample and d is the optical path length. The expression $\exp\left(\eta d|\psi(x_n,t)|^2\right) - 1$ accounts for the transient differential transmission signal $\Delta T/T$ that has been reported in experiments.[4,16] For small values of $\eta d|\psi(x_n,t)|^2$, which is typically true for wavepacket amplitudes in our simulations, the transient differential transmission signal is approximately $\exp\left(\eta d|\psi(x_n,t)|^2\right) - 1 \approx \eta d|\psi(x_n,t)|^2$, and the transient MSD expression becomes

$$\sigma^2(t) = \eta d \sum_{n=0}^{N-1} |\psi(x_n,t)|^2 \cdot (x_n - \langle x \rangle)^2, \tag{S64}$$

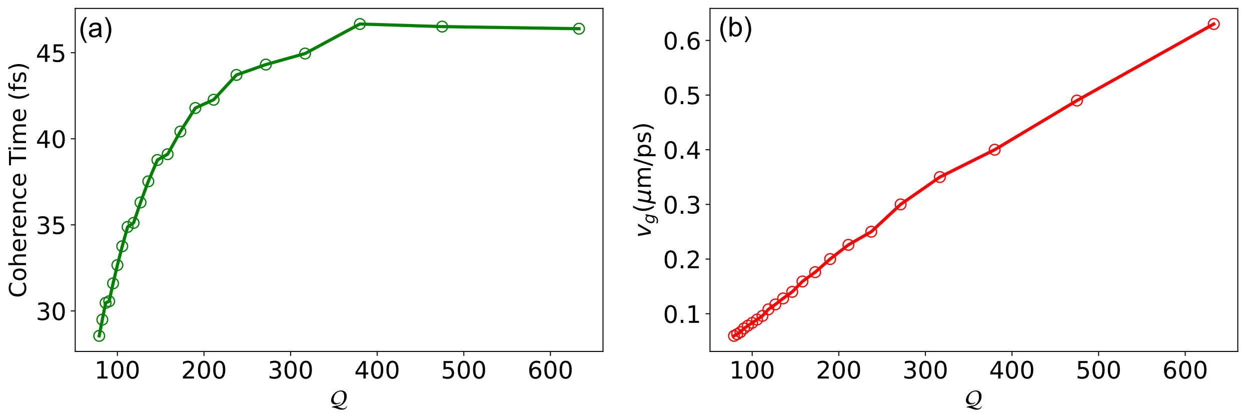

Figure S8: (**a**) Coherence loss time τ and (**b**) polariton group velocity v_g extracted with Eq. S62 using the transient MSD of the broadband UP excitation computed from Eq. S63.

which is identical to our expression in Eq. 9 from the main text *except* for a scaling factor of ηd.

Fig. S7 shows the transient MSD computed using Eq. S63 for a broadband UP and LP excitation. In our analysis, we set $\eta d = 0.00053$ to match the peak values of the transient MSD from simulations to the transient MSD measured in experiments.[16] We note that the overall trend for the transient MSD that we obtain from Eq. S63 is identical to the transient MSD computed from Eq. 9. The only difference is the absolute value of the MSD, which was in the μm^2 range using Eq. 9 and in the nm^2 range using Eq. S63. With the transient MSD computed from the transient absorption signals, we extract v_g and τ with Eq. S62 and plot these values with respect to cavity $\mathcal{Q}$-factor for the UP broadband excitation as shown in Fig. S8. We note that the coherence loss time extracted from our data is smaller than the coherence loss time reported in experiments, and we attribute it to the rapid increase and quenching of the UP transient MSD in our simulations. For v_g, we report values of 0.63 μm/ps for cavity $\mathcal{Q}$-factor of 633 to 0.0595 μm/ps for cavity $\mathcal{Q}$-factor of 79, which are consistent with the values reported in the experiments from Ref. 16.

References

(1) Mandal, A.; Taylor, M. A.; Weight, B. M.; Koessler, E. R.; Li, X.; Huo, P. Theoretical advances in polariton chemistry and molecular cavity quantum electrodynamics. *Chemical Reviews* **2023**, *123*, 9786–9879.

(2) Tichauer, R. H.; Feist, J.; Groenhof, G. Multi-scale dynamics simulations of molecular polaritons: The effect of multiple cavity modes on polariton relaxation. *The Journal of Chemical Physics* **2021**, *154*, 104112.

(3) Xu, D.; Mandal, A.; Baxter, J. M.; Cheng, S.-W.; Lee, I.; Su, H.; Liu, S.; Reichman, D. R.; Delor, M. Ultrafast imaging of polariton propagation and interactions. *Nature Communications* **2023**, *14*, 3881.

(4) Tichauer, R. H.; Sokolovskii, I.; Groenhof, G. Tuning the Coherent Propagation of Organic Exciton-Polaritons through the Cavity Q-factor. *Advanced Science* **2023**, *10*, 2302650.

(5) Nitzan, A. *Chemical dynamics in condensed phases: relaxation, transfer and reactions in condensed molecular systems*; Oxford university press, 2006.

(6) Huo, P.; Coker, D. F. Semi-classical path integral non-adiabatic dynamics: a partial linearized classical mapping Hamiltonian approach. *Mol. Phys.* **2012**, *110*, 1035–1052.

(7) Qiu, L.; Mandal, A.; Morshed, O.; Meidenbauer, M. T.; Girten, W.; Huo, P.; Vamivakas, A. N.; Krauss, T. D. Molecular polaritons generated from strong coupling between CdSe nanoplatelets and a dielectric optical cavity. *The Journal of Physical Chemistry Letters* **2021**, *12*, 5030–5038.

(8) Koessler, E. R.; Mandal, A.; Huo, P. Incorporating Lindblad decay dynamics into mixed quantum-classical simulations. *The Journal of Chemical Physics* **2022**, *157*, 064101.

(9) Harris, C. R. et al. Array programming with NumPy. *Nature* **2020**, *585*, 357–362.

(10) Cooley, J. W.; Tukey, J. W. An algorithm for the machine calculation of complex Fourier series. *Mathematics of computation* **1965**, *19*, 297–301.

(11) Press, W. H. *Numerical recipes 3rd edition: The art of scientific computing*; Cambridge university press, 2007.

(12) Mondal, M. E.; Vamivakas, A. N.; Cundiff, S. T.; Krauss, T. D.; Huo, P. Polariton spectra under the collective coupling regime. I. Efficient simulation of linear spectra and quantum dynamics. *The Journal of Chemical Physics* **2025**, *162*, 014114.

(13) Wales, D. J.; Doye, J. P. Global optimization by basin-hopping and the lowest energy structures of Lennard-Jones clusters containing up to 110 atoms. *The Journal of Physical Chemistry A* **1997**, *101*, 5111–5116.

(14) Lehoucq, R. B.; Sorensen, D. C.; Yang, C. *ARPACK users' guide: solution of large-scale eigenvalue problems with implicitly restarted Arnoldi methods*; SIAM, 1998.

(15) Voronycha, O.; Buraczewskia, A.; Matuszewski, M.; Stobińsk, M. Numerical modeling of exciton–polariton Bose–Einstein condensate in a microcavity. *Comp. Phys. Comm.* **2017**, *215*, 246–258.

(16) Pandya, R.; Ashoka, A.; Georgiou, K.; Sung, J.; Jayaprakash, R.; Renken, S.; Gai, L.; Shen, Z.; Rao, A.; Musser, A. J. Tuning the coherent propagation of organic exciton-polaritons through dark state delocalization. *Advanced Science* **2022**, *9*, 2105569.

(17) Chng, B. X.; Ying, W.; Lai, Y.; Vamivakas, A. N.; Cundiff, S. T.; Krauss, T. D.; Huo, P. Mechanism of Molecular Polariton Decoherence in the Collective Light–Matter Couplings Regime. *The Journal of Physical Chemistry Letters* **2024**, *15*, 11773–11783.

(18) Lai, Y.; Ying, W.; Huo, P. Non-Equilibrium Rate Theory for Polariton Relaxation Dynamics. *The Journal of Chemical Physics* **2024**, *161*, 104109.
